# Subnational estimates of factors associated with under-five mortality in Kenya: a spatio-temporal analysis, 1993–2014

**DOI:** 10.1136/bmjgh-2020-004544

**Published:** 2021-04-15

**Authors:** Peter M Macharia, Noel K Joseph, Benn Sartorius, Robert W Snow, Emelda A Okiro

**Affiliations:** 1 Population Health Unit, KEMRI-Wellcome Trust Research Programme, Nairobi, Kenya; 2 Centre for Tropical Medicine and Global Health, Nuffield Department of Medicine, University of Oxford, Oxford, UK; 3 Department of Health Metrics Sciences, School of Medicine, University of Washington, Seattle, WA, USA

**Keywords:** child health, geographic information systems, indices of health and disease and standardisation of rates, epidemiology, health services research

## Abstract

**Background:**

To improve child survival, it is necessary to describe and understand the spatial and temporal variation of factors associated with child survival beyond national aggregates, anchored at decentralised health planning units. Therefore, we aimed to provide subnational estimates of factors associated with child survival while elucidating areas of progress, stagnation and decline in Kenya.

**Methods:**

Twenty household surveys and three population censuses conducted since 1989 were assembled and spatially aligned to 47 subnational Kenyan county boundaries. Bayesian spatio-temporal Gaussian process regression models accounting for inadequate sample size and spatio-temporal relatedness were fitted for 43 factors at county level between 1993 and 2014.

**Results:**

Nationally, the coverage and prevalence were highly variable with 38 factors recording an improvement. The absolute percentage change (1993–2014) was heterogeneous ranging between 1% and 898%. At the county level, the estimates varied across space and over time with a majority showing improvements after 2008 which was preceded by a period of deterioration (late-1990 to early-2000). Counties in Northern Kenya were consistently observed to have lower coverage of interventions and remained disadvantaged in 2014 while areas around Central Kenya had and historically have had higher coverage across all intervention domains. Most factors in Western and South-East Kenya recorded moderate intervention coverage although having a high infection prevalence of both HIV and malaria.

**Conclusion:**

The heterogeneous estimates necessitates prioritisation of the marginalised counties to achieve health equity and improve child survival uniformly across the country. Efforts are required to narrow the gap between counties across all the drivers of child survival. The generated estimates will facilitate improved benchmarking and establish a baseline for monitoring child development goals at subnational level.

**Key questions**

**What is already known?**

- The recent under-five mortality (U5M) decline in Kenya has been uneven across subnational units and over time with widespread inequities. These inequities are likely to be associated with the uneven use of interventions, healthcare utilisation, distribution of resources and disease prevalence. Thus, subnational estimates of factors associated with child survival are required.

**What are the new findings?**

- Overall, the coverage of interventions increased between 1993 and 2014, however, the estimates were heterogeneous with widespread geospatial inequities at county-level over time.
- High heterogeneity and varied estimates between the factors ranging from low (less than 35%) for improved sanitation to high (over 65%) for childhood immunisations by 2014.
- Across almost all the factors, counties in Northern Kenya were systematically left behind with lower coverage, Central Kenya always had higher coverage while western and south-east counties had moderate coverage and higher HIV and malaria infection prevalence.

**What do the new findings imply?**

- Prioritised targeting in the marginalised Northern Kenya region during allocation of resources, finances, policy formulation and planning to increase coverage, reduce health inequities and improve child survival. Additional efforts to reduce malaria and HIV infection in western and coastal regions of Kenya are needed.
- Generated estimates will facilitate benchmarking between counties and form a key baseline for monitoring sustainable development goals and local targets at subnational level.
- Need to evaluate the contribution for each factor relative to U5M variation subnationally to aid in granular targeting.
- Need for additional data for monitoring coverage during the devolved health planning era in Kenya.

## Introduction

The planning and allocation of child health interventions to subnational areas with the greatest need is crucial in improving child survival equitably.^1 2^ To identify populations that are marginalised from healthcare access and preventive interventions, requires an evaluation of their coverage. This is necessary to achieve universal health coverage on the pathway to equitable improvements of child survival^3^ and ensure that no child is left behind and that resources do not go to waste on populations with the least need.^2^ This is enshrined within the sustainable development goals (SDGs) principle of *leaving no one behind and reaching the furthest behind, first*.^4 5^

Increased need for improved understanding of gaps in intervention coverage and other factors known to be associated with under-five mortality (U5M) at local health planning units has spurred improvements in the use of geocoded data from household sample surveys^6 7^ within advanced statistical modelling techniques.^8–11^ As a result, factors associated with U5M have been mapped at fine-scale spatial and temporal resolution across much of sub-Saharan Africa^6 10 12–19^ including Kenya.^6 10 16–23^ Previous approaches in Kenya have not always been comparable over time, nor have these studies harnessed all the available data to make predictions across the country’s subnational units required for decentralised health planning.^6 10 16–23^ Many of these studies have considered a few individual factors only, however, mapping all factors is fundamental for benchmarking of health systems performance across subnational units.^12–15^

Here, we leverage data from multiple sources including all available household sample surveys and population census to provide annual estimates of 43 factors known to be associated with changes in U5M at each of the 47 subnational counties used for decentralised health planning in Kenya.^24^ The generated estimates are used to express spatial and temporal inequities and elucidate areas of marginalisation for the periods between 1993 and 2014.

## Methods

### Country health context

Kenya’s healthcare system is pluralistic with both public and private healthcare facilities providing services. The service delivery is hierarchical with six tiers spanning between community level and tertiary facilities.^25^ The government has continually enhanced healthcare utilisation by ensuring healthcare services are affordable and accessible since independence through polices on user fee.^3 26–32^ Establishment of the Kenya Expanded Programme on Immunization (EPI) in 1980 introduced vaccines for six major killer diseases at the time including tuberculosis, polio, diphtheria, whooping cough, tetanus and measles. Between 2001 and 2014, yellow fever, hepatitis B and haemophilus influenza B type (*Hib*), pneumococcal conjugate, measles second dose and rotavirus vaccinations were added to the EPI schedule.^33^

Government-led campaigns such as the school feeding, *Malezi Bora* (good upbringing), baby-friendly initiatives at the hospital and community levels (Baby-Friendly Community/Hospital Initiative (BFCI and BFHI)) have addressed poor breastfeeding practices and improved nutrition among children.^34–36^ Initiatives to fight malaria targeted to children and mothers were intensified from 2000 through expanded, free delivery of insecticide-treated nets (ITNs),^37 38^ replacing failing malaria drugs with efficacious therapeutics,^39 40^ targeted indoor residual spraying (IRS) and intermittent preventive treatment in pregnancy (IPTp).^22 38^ Beginning 2000, there was an expansion of HIV prevention interventions including an increase in facilities offering prevention of mother-to-child transmission (PMTCT) interventions and increased uptake of antiretrovirals (ARVs) drugs.^41–43^

In 1996/1997, the Integrated Management of Childhood Illness was introduced aiming to improve the management of childhood illness such as diarrhoea, pneumonia, malaria, measles and malnutrition. It involves: (i) strengthening health worker skills in managing illnesses, (ii) strengthening health systems (drug availability, supervision, referral and Health Management Information System (HMIS)) and (iii) the improvement of family and community health practices through community involvement and awareness on measures to improve child health.^44^ After 2003, the millennium development goals created an impetus to improve child survival and led to a proliferation of reproductive, maternal, newborn, and child health policies, programmes and increased funding in Kenya.^45^

Kenya has made major strides in the promotion of child health through legal frameworks such as the children Act, vision 2030, Big Four agenda and a new constitution.^24 46^ Conversely, Kenya has been affected by several major disasters including floods, droughts, epidemics and post-election violence^47–51^ which are antagonistic to child survival. Online supplemental file 1 summarises both the health context and major disasters related to child survival in Kenya since independence.

10.1136/bmjgh-2020-004544.supp1 Supplementary data

**Figure 1:**
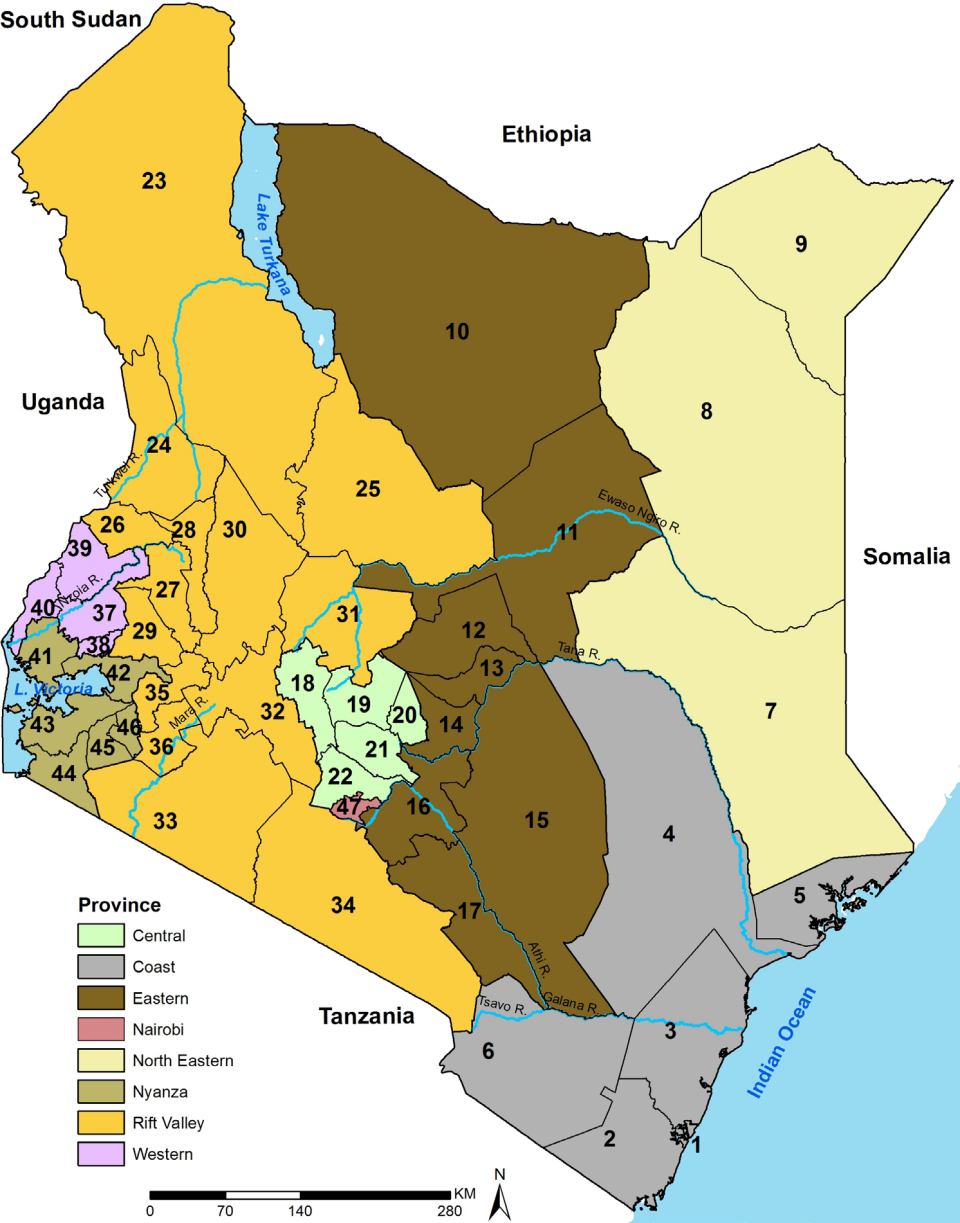
The map of Kenya showing 8 provinces (coloured) and the 47 subnational units (counties) as dark lines, water bodies and major rivers are shown in blue. Source: author. Coast province: Mombasa (1), Kwale (2), Kilifi (3), Tana River (4), Lamu (5), Taita Taveta (6); north eastern province: Garissa (7), Wajir (8), Mandera (9); eastern province: Marsabit (10), Isiolo (11), Meru (12), Tharaka Nithi (13), Embu (14), Kitui (15), Machakos (16), Makueni (17); central province: Nyandarua (18), Nyeri (19), Kirinyaga (20), Murang'a (21), Kiambu (22); Rift Valley province: Turkana (23), West Pokot (24), Samburu (25), Trans Nzoia (26), Uasin Gishu (27), Elgeyo Marakwet (28), Nandi (29), Baringo (30), Laikipia (31), Nakuru (32), Narok (33), Kajiado (34), Kericho (35), Bomet (36); western province: Kakamega (37), Vihiga (38), Bungoma (39), Busia (40); Nyanza province: Siaya (41), Kisumu (42), Homa Bay (43), Migori (44), Kisii (45), Nyamira (46); Nairobi province: Nairobi (47).

Following the promulgation of a new constitution in 2010 and the 2013 general elections, Kenya transitioned into a devolved system of government with a central government and 47 semi-autonomous county governments.^24^ The counties are now used for decentralised health planning^52^ and were adopted as the unit of analysis (figure 1). Under this system, the central Ministry of Health is mandated with policy-making and regulatory roles while allocation and managing healthcare resources and service provision is under the county governments.^53 54^

Subnational variations in U5M between 1965 and 2014 in Kenya have previously been described in detail in Macharia *et al*.^1^ Briefly, U5M was highly variable in space and time during the period under consideration (1993–2014). In 1993, 15 counties had U5M of ≥100 deaths per 1000 live births and were located in Western Kenya (Homa Bay, Migori, Siaya, Kisumu, Busia, Kakamega, Vihiga, Bungoma and Kisii), parts of coastal Kenya (Kilifi, Tana River, Lamu and Kwale), northern (Turkana, Garissa) and eastern (Kitui) regions. Five counties in central region (Kiambu, Embu, Murang’a, Nyeri and Nyandarua) and three in the neighbouring counties (Nakuru, Kajiado and Laikipia) had the highest probability of child survival, ≤50 deaths per 1000 live births in the same year.

During this period (1993–2014), 39 (83%) counties recorded U5M declines ranging between 1.6% in Kiambu and 58.3% in Mandera county. The counties with huge reductions (western and north eastern) had higher U5M in 1993 compared with counties with lower reductions. No county had U5M rates of ≤25 per 1000 live births, the target for 2030 for SDG 3.2 and by 2014, only three counties in western region (Migori, Homa Bay and Siaya) had U5M of ≥100 deaths per 1000 live births.

### Data

We accessed information from multiple household sample surveys and population censuses conducted since 1989 available from online data portals. These included the Integrated Public Use Microdata Series,^55^ Kenya National Bureau of Statistics,^56^ Multiple Indicator Cluster Surveys^57^ and Demographic and Health Survey.^58^ A survey was included if it contained at least one factor associated with U5M monitored over time, surveyed both in the rural and urban areas and devoid of reported data collection and quality concerns.

The assembled data were spatially misaligned over time due to changes in the number and geographical size of districts (subnational units before counties^1^) between 1989 and 2010 when county boundaries were formally defined.^24^ Spatially misaligned historical district boundaries were matched to the current county boundaries (figure 1) as described elsewhere.^1^ Table 1 summarises the data sources that were included in the analysis.

**Table 1:** Household sample surveys and population censuses undertaken since 1989 used in the analysis comprising six DHS, five MICS, three population censuses, two AIS, three MIS, two WMS and two KIHBS

Survey	Year	Number of counties	Number of households	Clusters/units	Women aged 15–49 years
DHS	1989	38	8173	393	7150
	1993	40	7950	520	7540
	1998	38	8380	536	7881
	2003	47	8561	400	8195
	2008/2009	47	9057	400	8444
	2014	47	36 430	1612	31 079
MICS	2000	42	9045	900	10 537
	2007	3	881	62	881
	2008	8	14 677	650	13 606
	2011	6	6828	300	5908
	2013/2014	3	3744	158	3348
Population Census	1989	47	224 861	36 979	238 027
	1999	47	317 106	61 921	345 647
	2009	47	243 858	96 253	934 904
AIS	2007	47	9691	402	5991
	2012	44	8035	371	7958
MIS	2007	43	6854	200	6111
	2010	47	6538	240	5749
	2015	47	6481	245	5394
WMS	1994	47	10 860	1172	13 385
	1997	41	10 873	1107	2484
KIHBS	2005/2006	47	13 390	1339	16 446
	2015/2016	47	21 773	2387	23 768

Table includes the number of counties covered, clusters and number of women of childbearing (15–49 years).

AIS, AIDS Indicator Survey; DHS, Demographic and Health Surveys; KIHBS, Kenya Integrated Household Budget Survey; MICS, Multiple Indicator Cluster Surveys; MIS, Malaria Indicator Surveys; WMS, Welfare Monitoring Surveys.

### Factors associated with U5M

Forty-three factors a priori known to be associated with U5M (table 2 and online supplemental file 2) were identified based on existing frameworks of child survival,^59–61^ relevance to Kenya’s child health priorities^52^ and data availability (table 1). The factors were defined following household sample surveys guidelines.^56–58^ The 43 factors were divided into 8 broad thematic areas including environmental, maternal, child, and household factors, infections, healthcare utilisation, child and maternal health interventions. Table 2 outlines the factors and their thematic groups with detailed definitions and the specific data sources outlined in online supplemental file 2.

10.1136/bmjgh-2020-004544.supp2 Supplementary data

**Table 2:** The factors associated with child survival and thematic groups as used in the current analysis

Group	ID	Variable
Environmental factors	1	Rural residency
	2	Precipitation
	3*	Enhanced vegetation index (EVI)
Maternal factors	4	Maternal education
	5	Maternal literacy
	6	Female headed households
	7	Short birth spacing
	8	Use of modern contraceptives
	9	High parity
Child factors	10	Underweight
	11	Wasted
	12	Stunted
	13	Breast fed within the first hour of birth
	14	Exclusive breast feeding
	15	Continued breast feeding
	16	Low birth weight (LBW)
Household factors	17	Poor household
	18	Improved sanitation
	19	Access to any form of a toilet
	10	Improved water
	21	Access to wells, borehole and piped water
Infections	22	HIV infection prevalence
	23	Malaria infection prevalence
Healthcare utilisation	24	At least one antenatal care visit (ANC1)
	25	At least four antenatal care visits (ANC4)
	26	Skilled birth attendance (SBA)
	27	Health facility deliveries (HFD)
	28	Diarrhoea treatment-seeking
	29	Fever/cough treatment-seeking
Child health interventions	30	BCG
	31	Three diphtheria–tetanus–pertussis vaccinations (DPT3)
	32	Three doses of polio (Polio3)
	33	Measles
	34	Fully immunised
	35	Oral rehydration salts (ORS use)
	36*	Vitamin A-children
	37*	Insecticide treated bed nets (ITN) use by children
	38*	Recommended antimalarial use
Maternal health interventions	39	Tetanus toxoid injection
	40*	Intermittent preventive treatment in pregnancy (IPTp 1)
	41*	IPTp 2
	42*	Iron supplement
	43*	Vitamin A-mothers

The definitions and respective data sources of factors are shown in online supplemental file 2.

*2003 is the baseline year because the corresponding factors were either not monitored or had not been rolled out.

The comparativeness of various sources of information considered in the current analysis is non-trivial (table 1) given the variations in period, approaches and tools used during data collection. To ensure comparativeness of the various data sources, we implemented several checks. First, we explored and assessed the responses collected per each factor under consideration across all household sample surveys and population censuses (table 1). We then adopted a definition (online supplemental file 2) that ensured identical meaning across all data sources to maintain temporal comparability and allow for tracking of changes over time. Second, using approaches outlined by Ngandu *et al*,^62^ we evaluated the effect of recall bias and missing data on estimates when combining Demographic and Health Surveys (DHS) and Multiple Indicator Cluster Surveys (MICS) data. The results were reasonably comparable using unmatched time lags for 3 years (DHS) and 2 years (MICS) and was adopted for our analyses based on a pragmatic balance between recall data and maintaining a large enough sample for county level estimates. Finally, the use of spatio-temporal methods described in the subnational modelling section allowed for smoothing of data points from the multiple sources.

### Subnational modelling

Coverage and/or prevalence for 39 factors were estimated using data defined in table 1 while 4 factors were available either as gridded surfaces or at aggregated geographical units. The four factors included HIV infection prevalence at the provincial level (figure 1) available from Kenya National AIDS Control Council (NACC),^63^ a temporal gridded malaria risk surface based on parasite prevalence,^22^ and temporal gridded surfaces of enhanced vegetation index (EVI) and precipitation.^64 65^ Thirty-five factors were available for the entire analysis period (1993–2014) while eight factors were available from 2003 since they were either rolled out or first monitored from early 2000 (table 2).

Prevalence estimates for each of the 39 factors (table 2) were computed while accounting for sampling design and/or survey weights by survey/census (table 1) at county level. All the household sample surveys (except DHS 2014) were designed to provide precise estimates at national and provincial levels and not powered to provide subnational county-level estimates. To predict across all subnational counties and during non-sampled years and combining raw estimates where more than one survey was conducted in a single year, a Bayesian spatio-temporal Gaussian Process Regression model^66 67^ with a heteroscedastic error component defined elsewhere^1^ was used (equation 1). In brief, the modelling framework accounts for large sampling variance and heterogeneity between surveys while exploiting spatio-temporal relatedness to increase predictive power (equation 1).

Spatio-temporal model for smoothing factors associated with U5M:

$$log\left\{\frac{Q_{ikt}}{1-Q_{ikt}}\right\}=\alpha +S_{kt}+Z_{kt}\;\;(1)$$

$Q_{ikt}$is the weighted proportion for each factor (table 2) for survey i (table 1), county k (n=47; figure 1) and year t (n=22 or 12 years; table 1, online supplemental file 2); α is the intercept, $S_{kt}$ is a spatio-temporal Gaussian process predicted by borrowing strength of information across surveys, counties and years with mean 0 and covariance function $\Sigma =\sigma^{2}\left[R_{S}⊗R_{T}\right]$. Where ⊗ is the Kronecker product, R_S_ and R_T_ are the spatial and temporal correlation matrices, respectively. R_S_ is modelled using a conditionally autoregressive (CAR) process while R_T_ is an autoregressive process of the first order. $Z_{kt}$ a Gaussian noise modelled with the variance taken to be the product of the log-transformed sample size from a given survey, county and year.^1^ Further details of the modelling framework are presented in online supplemental file 3 in Macharia *et al.* 1

10.1136/bmjgh-2020-004544.supp3 Supplementary data

The model for each factor was fitted using Markov chain Monte Carlo (MCMC) algorithm based on 10 000 posterior samples by county and year. The algorithm was iterated for 110 000 times and retained every 10th sample after a burn-in of 10 000 samples. Cross-validation was undertaken to assess the predictive performance of the model through a 10% random hold-out of the observed values. The observed and posterior predictions were used to compute the correlation, mean absolute error and root mean square error. The posterior distribution for each factor was summarised by its mean and 95% CIs for each year and county across the study period.

Analyses and data management were conducted in StataCorp 2014 (Stata Statistical Software: Release V.14) and R statistical software (V.3.4.1) while all the cartographies were done in ArcMap V.10.5 (ESRI, Redlands, CA, USA). While estimates were generated for every year between 1993 and 2014, maps were anchored at stable years with substantial data points corresponding to years when majority of the nation-wide sample surveys were conducted (1993, 1998, 2003, 2008 and 2014).

### Patient and public involvement

The study used secondary data only (table 1) that are publicly available through links and sources provided within the manuscript.^55–58^

## Results

The data assembled included 20 sample household surveys and 3 population censuses conducted after 1989 covering 870 county-years and 1.7 million women of childbearing age (table 1). The annual predictions spanned either the entire analysis period (1993–2014) for 35 (82%) factors or 12 years (2003–2014) for seven interventions that were introduced or first monitored from the early-2000s.

At the national level, at baseline (1993), child health interventions had high coverage (over 65%). Conversely, rates of healthcare utilisation were moderate (35%–65%) except at least one antenatal care visit (ANC1) which had a high coverage (91%). Both maternal health interventions and household-related factors had either moderate or low coverage (table 3). The coverage of nutritional factors was highly heterogeneous. For example, breastfeeding factors spanned between low (exclusive breast feeding), moderate (breast fed within 1 hour of birth) to high (continued breast feeding) coverage while the prevalence of malnutrition ranged between 7% (acute-malnutrition or wasting) and 40% (chronic-malnutrition or stunting) with underweight (elements of both stunting and wasting) being 19% (table 3). The coverage/prevalence of maternal factors was variable ranging from low (contraceptive use) to high (maternal literacy). The infection prevalence of malaria (24%) and HIV (9%) were high. Table 3 shows the estimate of all factors in 1993 and 2014.

**Table 3:** The national coverage and or prevalence of the factors associated with child survival in 1993 and 2014 and change between the two time points

Thematic group	ID	Factors associated with under-five mortality	Estimate (95% CI)		Change (95% CI)	
			1993	2014	Percentage	Absolute
Environmental factors	1	Rural residency	84.2 (83.5 to 84.9)	58.0 (57.7 to 58.8)	−31.3 (−31.7 to −30.9)	−26.4 (−26.8 to −26.0)
	2	Precipitation	35 455.97 mm	43 866.85 mm	19.2	8410.9
	3	EVI	0.33	0.31	−5.5	0.02
Maternal factors	4	Maternal education less than pry school	57.7 (56.4 to 59.0)	36.38 (35.6 to 37.2)	−36.9 (−37.5 to −36.3)	−21.3 (−21.9 to −20.7)
	5	Maternal literacy	76.7 (75.5 to 77.8)	84.1 (83.5 to 84.7)	9.6 (9.1 to 10.2)	7.4 (6.9 to 7.9)
	6	Female household head	33.6 (32.7 to 34.5)	32.2 (31.7 to 32.7)	−4.2 (−4.6 to −3.8)	−1.4 (−1.8 to −1.0)
	7	Short birth interval	25.2 (24.1 to 26.2)	16.9 (16.4 to 17.5)	−49.1 (−49.4 to −48.8)	−8.3 (−8.6 to −8.0)
	8	Modern contraceptives use	20.8 (20.0 to 21.5	39.5 (38.7 to 40.3)	90.8 (90.5 to 91.1)	18.8 (18.8 to 19.1)
	9	High parity	35.9 (35.0 to 36.8)	25.3 (20.8 to 21.17)	−30.1 (−30.5 to −29.7)	−10.8 (−11.2 to −10.4)
Child factors	10	Underweight	18.9 (18.0 to 19.9)	10.6 (10.2 to 11.1)	−43.9 (−44.3 to −43.5)	−8.3 (−8.7 to −7.9)
	11	Wasted	6.8 (6.2 to 7.4)	4.1 (3.8 to 4.4)	−39.7 (−39.9 to −39.5)	−2.7 (−2.9 to −2.5)
	12	Stunted	40.0 (38.8 to 41.2)	25.8 (25.2 to 26.5)	−35.5 (−36.0 to −35.0)	−14.2 (−14.7 to −13.7)
	13	Breast fed within first hour of birth	55.5 (54.1 to 56.8)	62.8 (61.8 to 64.1)	13.2 (12.3 to 14.1)	7.3 (6.4 to 8.2)
	14	Exclusive breast feeding	17.6 (14.8 to 20.4)	60.9 (57.5 to 64.3)	246.0 (245.1 to 246.9)	43.3 (42.4 to 44.2)
	15	Continued breast feeding	91.6 (90.2 to 92.9)	87.9 (85.2 to 90.1)	−4.0 (−5.7 to −2.3)	−3.7 (−5.4 to −2.0)
	16	Low birth weight	8.7 (7.7 to 9.6)	7.6 (6.9 to 8.2)	−12.6 (−13.1 to −12.1)	−1.1 (−1.6 to −0.6)
Household factors	17	Poor household	38.4 (37.4 to 39.3)	34.7 (34.2 to 35.2)	−9.6 (−10.0 to −9.2)	−3.7 (−4.1 to −3.3)
	18	Improved sanitation	8.4 (8.0 to 8.5)	17.6 (17.2 to 18.0)	109.5 (109.2 to 109.8)	9.2 (8.9 to 9.5)
	19	Improved and intermediate sanitation	83.2 (82.5 to 83.9)	90.1 (89.8 to 90.4)	8.3 (8.0 to 8.6)	6.9 (6.6 to 7.2)
	20	Improved water	29.9 (28.1 to 30.5)	43.6 (43.1 to 44.1)	45.8 (45.4 to 46.2)	13.7 (13.3 to 14.1)
	21	Improved and intermediate water	51.4 (50.5 to 52.4)	63.4 (62.9 to 63.8)	23.3 (22.9 to 23.7)	12.0 (11.6 to 12.4)
Infections	22	HIV	9.16	5.01	−82.8	−4.2
	23	Malaria	23.9 (16.1 to 34.8)	4.7 (3.6 to 8.4)	−80.3	−19.2
Healthcare utilisation	24	ANC1	91.4 (90.8 to 92.1)	95.6 (95.3 to 95.9)	4.6 (4.2 to 5.0)	4.2 (3.8 to 4.6)
	25	ANC4	62.4 (61.1 to 63.6)	56.3 (55.4 to 57.2)	−9.8 (−10.6 to −9.2)	−6.1 (−6.8 to −5.4)
	26	Skilled birth attendance	42.1 (42.0 to 44.3)	63.5 (62.8 to 64.3)	50.8 (50.1 to 51.5)	21.4 (20.7 to 22.1)
	27	Health facility births	41.6 (40.3 to 42.9)	63.0 (62.2 to 63.8)	51.4 (50.7 to 52.1)	21.4 (20.7 to 22.1)
	28	Diarrhoea treatment-seeking	39.2 (36.3 to 42.0)	57.8 (56.0 to 59.6)	47.4 (45.9 to 48.9)	18.6 (17.1 to 20.1)
	29	Fever treatment-seeking	46.0 (44.4 to 47.6)	72.8 (71.7 to 73.9	58.3 (57.3 to 59.3)	26.8 (25.8 to 27.8)
Child health interventions	30	BCG	96.2 (95.2 to 97.1)	96.7 (96.1 to 97.2)	0.5 (0.1 to 1.0)	0.5 (0.1 to 1.0)
	31	DPT3	86.7 (85.0 to 88.4)	90.1 (89.1 to 91.1)	3.9 (3.1 to 4.7)	3.4 (2.6 to 4.2)
	32	Polio3	85.5 (83.7 to 87.2)	90.9 (90.0 to 91.8)	6.3 (5.5 to 7.1)	5.4 (4.6 to 6.2)
	33	Measles	83.6 (81.8 to 85.8)	87.1 (86.0 to 88.1)	4.2 (3.3 to 5.1)	3.5 (2.6 to 4.4)
	34	Fully immunised	78.1 (76.0 to 80.2)	78.5 (77.5 to 80.1)	0.5 (−0.6 to 1.6)	0.4 (−0.7 to 1.5)
	35	ORS use	30.8 (28.0 to 33.5)	54.7 (52.8 to 56.5)	77.6 (76.0 to 79.2)	23.9 (22.3 to 25.5)
	36	Vitamin A-children*	34.1 (32.8 to 35.5)	67.8 (57.1 to 68.5)	98.8 (98.3 to 99.3)	33.7 (33.2 to 34.2)
	37	ITN use by children in malarious areas*	6.2 (5.4 to 7.0)	61.9 (61 to 62.8)	898.4 (897.7 to 899.1)	55.7 (55.0 to 56.4)
	38	Antimalarial use in malarious areas*	13.9 (12.0 to 15.9)	33.5 (31.9 to 15.2)	141.0 (139.8 to 142.2)	19.6 (18.4 to 20.8)
Maternal health interventions	39	Tetanus toxoid injection	51.8 (50.2 to 53.3)	51.5 (49.3 to 52.0)	−0.6 (−1.5 to 0.3)	−0.3 (−1.2 to 0.6)
	40	IPTp 1 in malarious areas*	13.3 (11.6 to 15.1)	41.2 (39.9 to 42.4)	210.5 (209.7 to 211.3)	27.9 (27.1 to 28.7)
	41	IPTp 2 in malarious areas*	5.6 (4.4 to 6.8)	25.7 (24.6 to 26.8)	358.9 (358.4 to 359.4)	20.1 (19.6 to 20.6)
	42	Iron supplement*	46.1 (44.5 to 47.6)	69.8 (68.7 to 70.9)	51.4 (50.4 to 52.4)	23.7 (22.7 to 24.7)
	43	Vitamin A-mothers*	14.3 (13.2 to 15.3)	54.5 (53.3 to 55.6)	281.1 (280.4 to 281.8)	40.2 (39.5 to 40.9)

*2003 is the baseline year because the corresponding factors were either not monitored or had not been rolled out. The four indicators without CI were externally sourced.

ANC1, one antenatal care visit; ANC4, four antenatal care visits; DPT3, three diphtheria–tetanus–pertussis vaccinations; EVI, enhanced vegetation index; IPTp, intermittent preventive treatment in pregnancy; ITN, insecticide-treated net; ORS, oral rehydration salts.

Nationally, all the factors had either an increase in the coverage of intervention or a decline in the infection prevalence between 1993 and 2014 except four factors (female-headed households, continued breast feeding, at least four antenatal care visits (ANC4) and tetanus toxoid injection) which showed minimal change. The percentage change in each of the four factors was less than 10%. The absolute percentage change was heterogeneous ranging from 1% to 898% across the 43 factors (table 3). Eighteen factors (42%) had an absolute percentage change of less than 25% while seven factors (16%) had a change of over 100%. Table 3 shows the percentage changes highlighting those that stagnated (red), had small (light green), moderate (mild green) or sizeable improvements (dark green).

By 2014, most of the factors had coverage of over 35%. For example, breastfeeding indicators, most maternal factors, household factors (except improved sanitation), maternal health interventions (except antimalarials and IPTp) and healthcare utilisation rates, all had moderate to high coverage (table 3).

**Figure 2:**
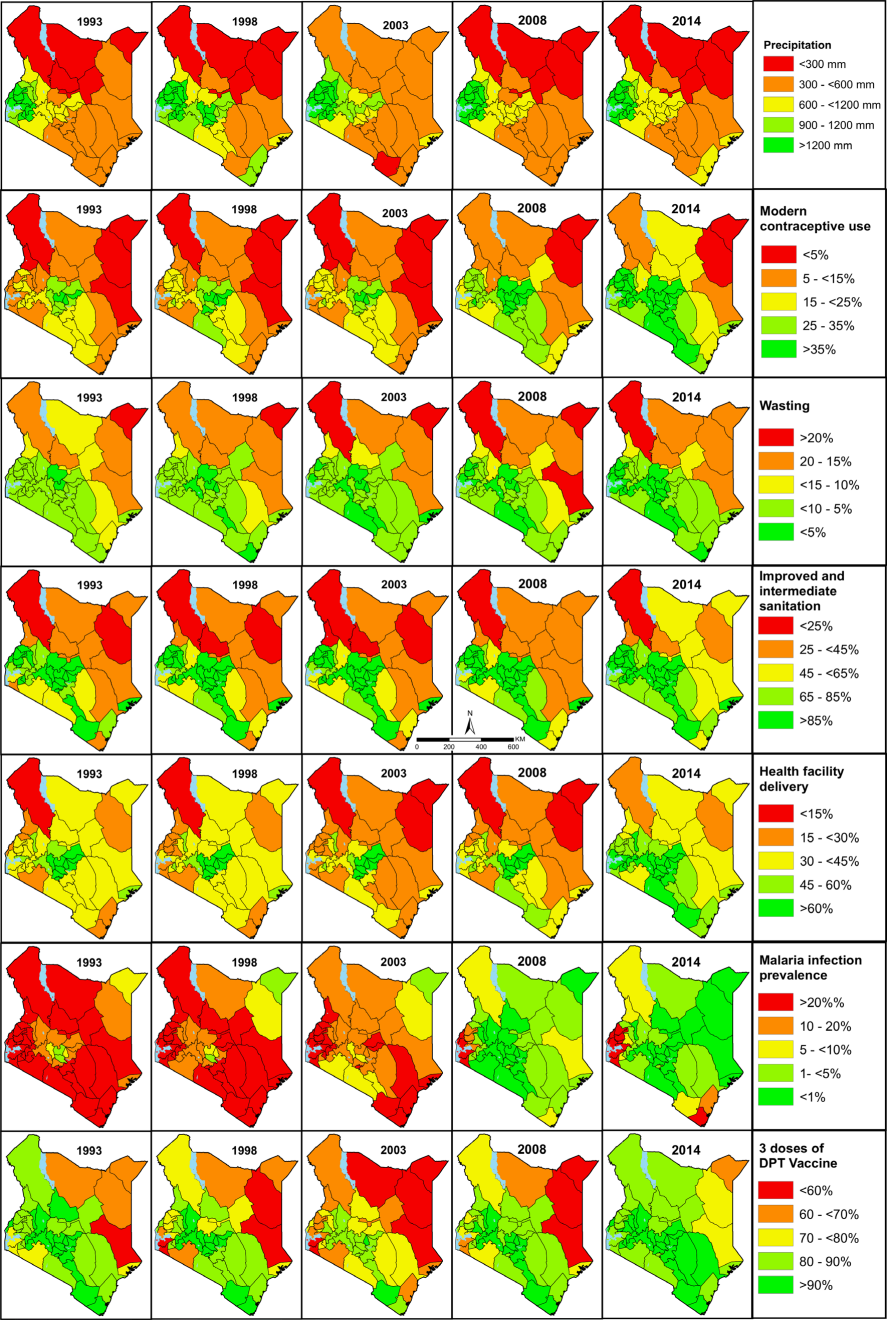
A subset of seven factors associated with child survival including precipitation, use of modern contraceptives, wasting, a combination of intermediate and improved sanitation, health facility delivery, malaria infection prevalence and three doses of diphtheria–tetanus–pertussis (DPT) vaccine. The factors are classified into five classes colour-coded from red (low coverage or high disease prevalence) to green (high coverage or low disease prevalence). The rest of the factors are presented in online supplemental file 3. Source: author.

**Figure 3:**
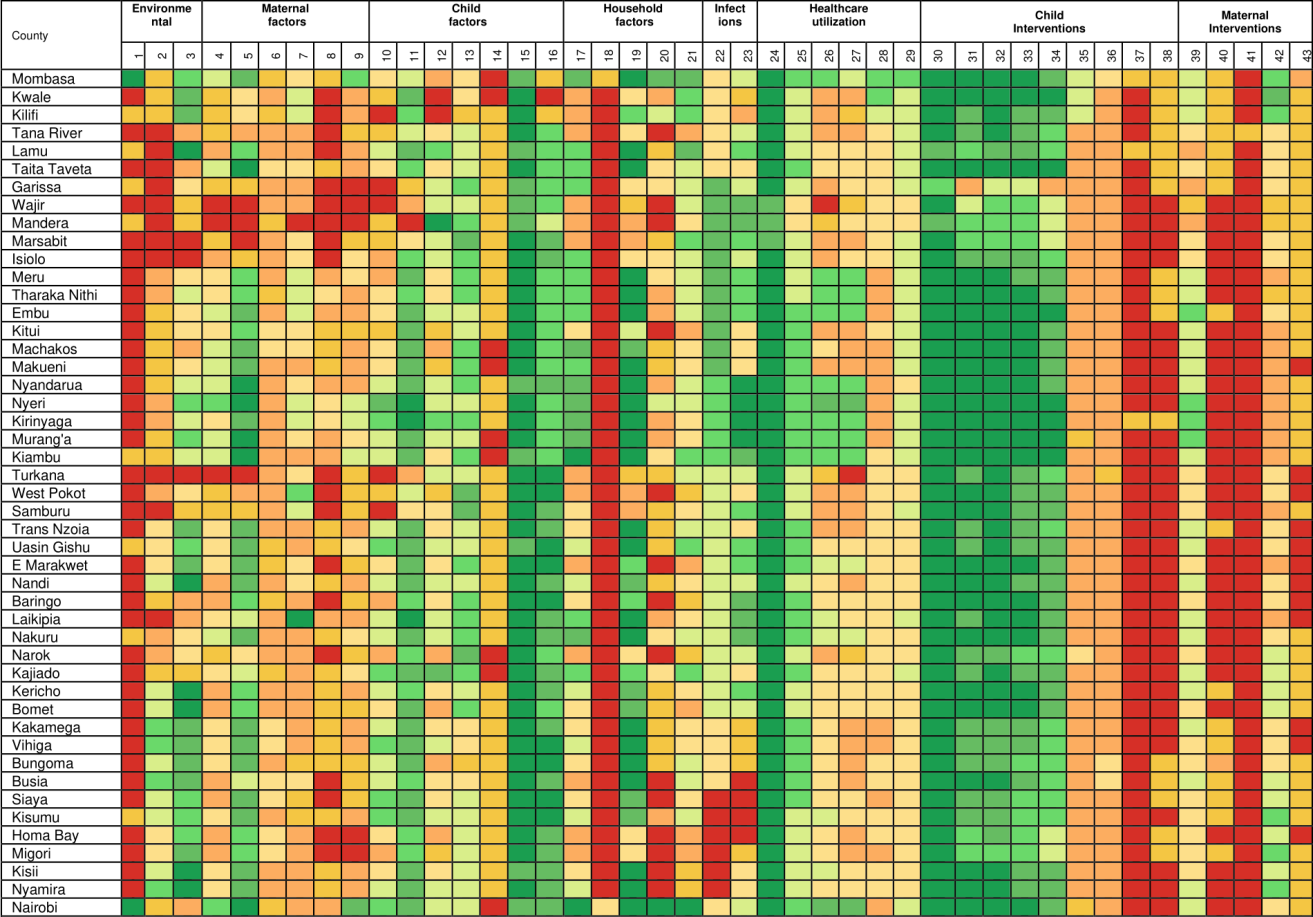
Ranking of factors associated with child survival in 1993 from low intervention coverage or high disease prevalence (red) to high intervention coverage or low disease prevalence (green). The second row are the 43 factors (table 2) while online supplemental file 3 has the actual values. Colour codes can be compared within an indicator across the 47 spatial units in 1993 and 2014 but not between factors. Source: author. An octile ranking was used to divide each factor into eight equal classes from <12.5% to ≥87.5% for interventions whose coverage varied from 0% to 100%. For factors whose coverage was not expected to range between 0% and 100% (such as nutrition status), they were first rescaled to 0%–100% and then divided into octiles.

**Figure 4:**
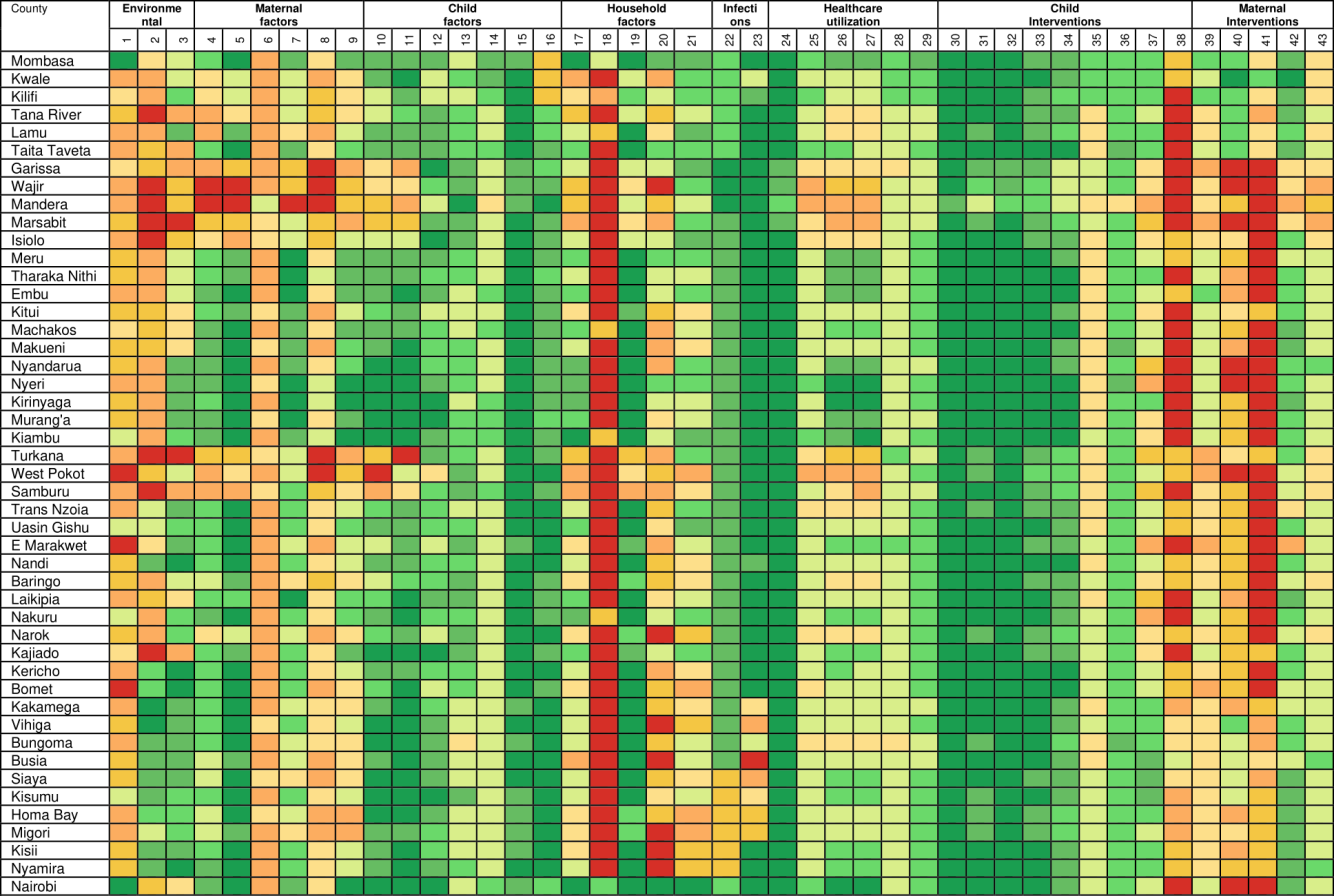
Ranking of factors associated with child survival in 2014 from low intervention coverage or high disease prevalence (red) to high intervention coverage or low disease prevalence (green). The second row are the 43 factors (table 2) while online supplemental file 3 has the actual values. Colour codes can be compared within a factor across the 47 spatial units in 1993 and 2014 but not between factors. Source: author. An octile ranking was used to divide each factor into eight equal classes from <12.5% to ≥87.5% for interventions whose coverage varied from 0% to 100%. For factors whose coverage was not expected to range between 0% and 100% (such as nutrition status), they were first rescaled to 0%–100% and then divided into octiles.

National estimates mask important subnational county differences in the coverage or prevalence of the factors associated with U5M over time. The subnational estimates for all the factors across the study are presented in online supplemental file 3 while a subset of seven factors across seven themes are presented in figure 2. Further, figure 3 (1993) and figure 4 (2014) show scaled heat plots of all 43 factors and the 47 counties across the study period representing the prevalence and coverage estimates. Overall, across the continuum of all factors, intervention coverage was lower and disease infection prevalence higher in 1993 compared with 2014 (figures 2–4 and online supplemental file 3).

The reduction in infection prevalence and the increase in intervention coverage was characterised by epochs of improvement, deterioration and reversals in gains made. The improvements were observed mainly after 2008 through to 2014 largely as a result of counties in Western Kenya and those neighbouring central parts catching up with the Central Kenya counties while counties in the northern parts of Kenya showed little improvements. Overall, the improvement phase (2008–2014) was preceded by a period of deterioration and stagnation between the late-1990s to early-2000s while the early-1990s was characterised by moderate coverage of most interventions (figures 2–4 and online supplemental file 3).

Despite the increase in intervention coverage and reduction in disease infection prevalence across factors and counties, there were notable exceptions. Instances, where coverage decreased by more than 5% in several counties included; a reduction in access to safe water, ANC4 coverage, use of recommended antimalarial medicine, tetanus toxoid vaccination coverage and an increase in the proportion of poor households. The counties most affected by these reversals were mainly located in Northern Kenya (Garissa, Wajir, Mandera, Marsabit, Turkana, West Pokot, Samburu) and partly western region (Trans Nzoia and Vihiga) and along the Indian ocean (Lamu) (figures 2–4 and online supplemental file 3).

The coverage of interventions and prevalence between factors was highly variable across the years. For example, there was a huge difference between the coverage of vaccinations such as three diphtheria–tetanus–pertussis vaccinations (DPT3) (mainly over 60% across counties) compared with access to modern contraceptives (mainly below 40% across all counties). Similarly, the usage of recommended antimalarial medicine and improved sanitation were always less than 20% compared with the coverage of ANC1 which was above 75% in a majority of the counties over the entire period (figures 2–4 and online supplemental file 3).

More prominent was the consistent colocation of disadvantaged counties over time for almost all factors. Counties in Northern Kenya had lower coverage for almost all maternal and child health (MCH) interventions, poor health utilisation rates, lower coverage of household factors and higher disease prevalence during the analysis period (online supplemental file 3). However, HIV and malaria infection prevalence were lower in Northern Kenya. Likewise, there was consistent colocation of counties that performed better (Central Kenya) from 1993 to 2014. In western and south-east counties, the coverage of interventions and disease prevalence were moderate except high infection prevalence of HIV and malaria (figures 2–4 and online supplemental file 3). The validation statistics showed a fair agreement between the observed and predicted estimates for a majority of the factors (82%) with a correlation coefficient above 0.6 (online supplemental file 4).

10.1136/bmjgh-2020-004544.supp4 Supplementary data

## Discussion

The compilation of a large database of household sample surveys, population census and other opportunistic sources allowed for the evaluation of trends, variations and changes in 43 factors associated with child survival at subnational county-level over two decades in Kenya. The geospatial framework applied harmonises previous approaches^6 10 16–23^ that have not always been comparable. The results depict substantial but heterogeneous gains in the provision and scaling up of MCH interventions between 1993 and 2014. The coverage ranged between suboptimal to moderate levels illustrating widespread disparities and inequities in the continuum of child and maternal healthcare. The trends, variations and changes observed, over time, especially between 2008 and 2014, can be linked to important initiatives and programmes in Kenya as summarised in the country health context and online supplemental file 1.

By 2014, two in every three children received child health interventions except for access to recommended antimalarial medicines (table 3). These include all childhood immunisations, oral rehydration salts, ITNs and vitamin A supplements. Childhood immunisations have been offered free of charge and probably why their coverages have been high over time. However, the proportion of fully immunised children stagnated (0.5% overall change) possibly due to those who do not receive timely immunisation or drop out before completing their immunisation schedule.^12 68–70^ Demand and supply challenges such as spatial access, health workforce, stockouts, cost of transportation and cold chain could also have hampered vaccination uptake especially in Northern Kenya where coverage was lower.^70–75^ Child and maternal survival interventions such as supplements have been delivered through the *Malezi Bora* initiative since 2007, a health facility-based delivery system migrating away from the previous door-to-door approach to reduce implementation cost.^35^ This might have also encouraged the uptake of immunisations. However, the coverage of maternal interventions was slightly lower than that of childhood interventions.

ITNs were only limited to the private and special project-based distributions until 2000 when they were partially subsidised through to 2004 followed by high subsidies and delivered via MCH clinics. After 2006, ITNs were available free of charge through routine delivery and mass delivery in 2006, 2008, 2011/2012, 2014 and 2015.^37 38^ Increase in coverage of ITNs among children coincides with these efforts (online supplemental file 3, figures 3 and 4). The regions with high malaria prevalence (counties in western, coastal and partly in the Kenyan highlands) have benefited from targeted ITN distribution and historical clinical trials which might explain their higher coverage relative to other regions.^38^ The low coverage of recommended antimalarial medicine might be due to frequent changes in first-line treatment of uncomplicated malaria; from chloroquine to the long half-life, single-dose sulfadoxine-pyrimethamine (SP) in 1998 which was later changed to artemisinin-based combination therapy in 2006.^38–40^

The period of greatest decline in malaria risk occurred prior to the scaling of ITNs and coincided with a period of use of infective drugs. The decline could be linked to the widespread availability of SP and its long half-life providing prophylaxis after single-dose administration.^76^ On the other hand, increasing HIV infection prevalence in the 1990s (online supplemental file 3) led to the establishment of NACC that put measures that coincided with HIV decline. Since 2000, PMTCT, paediatric HIV programmes, ARVs uptake, testing and behavioural change campaigns have increased steadily.^41–43 45 77–81^

Increase in healthcare utilisation rates can be linked to policies on user fees. The health voucher programme for maternity services (2006–2016), the abolishment of delivery fees (2007), free maternity services (2013) and suspension of user fee (1990).^3 26–32^ From 1991, user fees charged for services such as drugs and laboratory services hampered utilisation.^82–85^ This led to the removal of user fees at dispensaries and health centres (except a for registration fee) in 2004^3 26^ with a health sector services fund introduced in 2010 to compensate for the removal of user fee at dispensaries and health centres.^26^ Before the suspension of user fees, antenatal care utilisation declined in all counties with Mandera, Wajir, Marsabit, Garissa, Isiolo, Tana River and Turkana counties witnessing larger declines between 1993 and 2003 (online supplemental file 3). Similar trends were observed for other markers of healthcare utilisation such as institutional deliveries. However, an immediate shift in utilisation trends, declining in the 1990s (eg, institutional deliveries) especially in northern counties (Mandera, Isiolo, Garissa, Wajir, Marsabit, Samburu, Laikipia, Baringo and West Pokot) was witnessed from 2006 after the new policies on user fees (online supplemental file 3).

Long physical distances to point of care affect utilisation rates negatively, however, in Kenya, over time, the number of health facilities has increased reducing the travel time needed to seek care.^73–75^ Therefore, it is plausible that the quality and availability of services offered at points of care might be major factors relative to the physical distance in influencing utilisation rates in some parts of Kenya. The stagnation of ANC4 rates and some of the immunisations could be because they require multiple contacts with the health system, inadequate staff or poor adherence to treatment guidelines.^12 21^ However, counties in Northern Kenya, have always had poor physical access to healthcare services hence lower utilisation rates. In 2003, only 18% of the total population was within the recommended distance of a health facility (5 km radius) improving marginally to 29% in 2008 compared with national averages of 71% and 89%, respectively.^74^ For example, mean travel time of up to 120+ minutes was significantly associated with poor immunisation outcomes only in Northern Kenya (Isiolo and Marsabit).^71^

Low breastfeeding coverage and high malnutrition levels continue to be a public health concern for Kenya with moderate gains witnessed across time.^86–89^ The small improvements can partly be associated with breastfeeding initiatives (BFCI and BFHI) both at the hospital and community level,^36^ food fortification, micronutrients, nutritional campaigns, school and community level initiatives (online supplemental file 1). However, the number of children receiving the minimum acceptable diet was low and declined over time.^90–93^ The piloting and demonstration of BFCI in parts of Siaya (Bondo) and Meru (Igembe North) counties in 2011 showed increased likelihood of participating in ANC, institutional deliveries and initiating breast feeding within an hour of birth^94^ and appears to be linked to broad increases in coverage (online supplemental file 3). Following the demonstration, BFCI was included in the National Nutrition Action Plan and prioritised as a *high impact nutrition intervention*.^94^

The use of modern contraceptive increased by over 90%, however, despite this improvement by 2014, only 40% of the women in need of contraception were covered (table 3). Family planning reduces closely spaced births, ill-timed births and high parity and might explain why there was a moderate reduction in short birth interval and high parity^95–97^ (online supplemental file 3). There have been several efforts and initiatives to improve the coverage of family planning by addressing drivers of the slow progress.^98–102^ For, example, The government committed to increase the budget allocated for family planning services in 2012 which may have led to the achievement of 2020 target (58% coverage) and is now focusing on equitable access subnationally.^103 104^ The marked regional heterogeneities in contraceptive use (online supplemental file 3) have been linked with socioeconomic and cultural environment^102^ and possibly the regional initiatives addressing areas with lower coverage. For, example, after the launch of *AMUA* project (a social franchise) to provide family planning among under-served communities led to improved contraceptive use in the focus counties of western and coastal parts of Kenya between 2003 and 2008 (online supplemental file 3).^105^

The coverage of both improved sanitation and access to clean water at household level has remained low. The poor coverage has been associated with low education attainment, living in rural areas and poverty.^106^ The government of Kenya committed to focus on the poorest, eliminate open defecation by 2030 and to invest 0.5% of its gross domestic product by 2020 to sanitation.^107^ However, currently (2019), only a third of the households have access to piped water and 8.2% do not have access to any sanitation facility.^108^ Minimal improvement in household wealth (online supplemental file 3) compounded the already dire need for access for safe and clean water and improved sanitation.

Historical (1993–2013) county level data on subnational policy and their implications are scarce for periods before 2013 when the devolved government was incorporated; thus, county level discourse is based on exemplar and limited information. At the county level, the coverage of interventions was disproportionately distributed over time; the coverage ranged between high to acutely low in disadvantaged and marginalised areas. Overall, counties in Central and Western Kenya had a moderate to high intervention coverage while counties in Northern Kenya were marginalised across the entire study period.

Northern Kenya is predominately arid and semi-arid (ASAL) with a low amount of rainfall and vegetation (online supplemental file 3) associated with reduced yields from rain-fed agriculture, persistent food insecurity and lack of green pastures for livestock.^109 110^ They have challenges in accessing clean water and improved sanitation, low education attainment and more poor households, consequently, malnutrition is high in this region.^106 110^ Healthcare utilisation rates are low due to poor infrastructure and limited geographical access, conflict and insecurity hence low immunisation rates and use of modern contraceptives.^71 73 74 101 111^ However, in this region, religion and cultural beliefs might be a stronger determinant of contraception use in comparison to poverty and lack of access.^112–114^ In view of historical and economic similarities between regions in Kenya, six economic blocs were formed. Among them is the Frontier Counties Development Council consisting of ASAL counties mainly in Northern Kenya (Lamu, Tana River, Garissa, Wajir, Mandera, Marsabit, Isiolo, Turkana, Samburu and West Pokot) which aims to enhance socioeconomic development and sustainable development through better cooperation through projects such as livestock strengthening.^115^ These counties can further harness the assembled data to gain better insights on trends for informed decision making.

Conversely, the counties in Central Kenya with the higher coverage historically have higher agricultural productivity, lower rates of disease, better access to education, clean water and quality sanitation. Spatial access to healthcare is better in most parts of these counties, hence, better healthcare utilisation rates, access to vaccinations and supplementations.^71 74 75 116^ The infection prevalence of HIV and malaria have declined across much of the country but remain high in Western Kenya and parts of south-east (online supplemental file 3). This has led to targeted interventions, for example, restricting IPTp to high prevalence areas, the pilot introduction of RTSS vaccine, and focused efforts to increase coverage of ITNs, IRS larval source management^117^ and increased attention to HIV preventative interventions^43 118–121^ in these two regions.

In this paper, we have not attributed the changing coverage of interventions and infection prevalence variations to disparities in U5M.^1^ However, preliminary analysis, show that counties with high U5M (western and coastal region) were characterised with high HIV and malaria prevalence. Central region with low infection prevalence and higher coverage of interventions had lower U5M although smaller upsurges. Northern region with low HIV and malaria prevalence but constrained by low access to interventions, poor healthcare utilisation limited access to clean and safe water and sanitation had moderate U5M. The correlation coefficient between these factors and U5M (online supplemental file 5) were statistically significant for all but three factors. Malaria prevalence and early infant breast feeding had moderate correlation.^122^ This underpins the need for a rigorous examination of the impact of these factors on driving trends in U5M across the 47 counties. Such evidence would be important for the health planners and policy makers and for targeted resource allocation.

10.1136/bmjgh-2020-004544.supp5 Supplementary data

The estimates generated in this analysis have important implications on existing efforts to improve child survival across Kenya under the decentralised governance structure while *leaving no one behind and reaching the farthest behind, first*. County planners can gain insights on coverage and trends to facilitate prioritisation. For example, by 2014, Mandera’s coverage of ANC1, BCG and polio vaccines were ≥70% while improved sanitation and contraceptive use were ≤3% justifying additional funds and prioritisation for the latter factors. These estimates provide opportunities for benchmarking across counties where localised initiatives which have been shown to successfully improve coverage and in reduce disease prevalence.^12–15^ The success of *Afya Uzazi* (healthy parenthood) programme in Baringo and Nakuru counties in improving access to quality health services by targeting family planning, pregnancies and deliveries, can be a benchmarking point for neighbouring counties.

At the national level, the government through *The Commission on Revenue Allocation* can leverage on the generated estimates when distributing national-level resources to 47 counties. The commission uses a weighted average of key factors including health indicators and can leverage on these estimates to better disaggregate the differences between counties. Further, counties in Northern Kenya should be targeted and prioritised during resources allocation and policy formulation to increase intervention coverage while more concerted efforts should be directed to western and coastal parts of the country to lower HIV and malaria infection prevalence. Various divisions such immunisation, national malaria control programme, human nutrition and dietetics unit within the national Ministry of Health can use the estimates as baseline to evaluate the impact of interventions that had been rolled out. This evaluation can be extended to include international development partners such as the GAVI the vaccine alliance to evaluate the impact of their funding or justify support for immunisation outreach programme. The findings should also form a key baseline for monitoring SDGs indicators proposed under the Inter-Agency and Expert Group on SDG Indicators^123^ and county-specific targets as outlined in each county blue print, the County Integrated Development Plan for the 47 county governments.^124^ The estimates form a key input in epidemiological studies of child survival across Kenya and these will be made available through a data visualisation web portal. Finally, the modelling framework can be applied to update the subnational estimates and evaluate progress as new data sources becomes available such as the recently concluded population census in Kenya (2019).^55 108^

We compared our estimates with previous estimates generated from similar and comparable studies that computed the prevalence of wasting, stunting and underweight, access to improved water sources, coverage of DPT3 (for the period 2000–2014)^125–127^ and skilled birth attendance (SBA) (for the period 2014)^128^ (online supplemental file 6). The estimates were highly correlated and generally with good concordance, identifying and ranking nearly all similar counties with the highest coverage (or lowest disease prevalence) and those with lowest coverage (or highest disease prevalence) (online supplemental file 6). The slight differences observed with reference to SBA are likely due to more data used in the current study within a spatio-temporal model without covariates relative to fewer data sources within a spatial model with covariates used in the comparator.^128^

10.1136/bmjgh-2020-004544.supp6 Supplementary data

### Limitations

There are several caveats to this analysis. Tracking of coverage estimates beyond 2014 was not possible due to lack of data post-2014 limiting the number of the possible applications. Despite interpolating in space and time, household surveys are limited as they are conducted every three to five years powered for precise estimates at provincial level, thus they are not an alternative to quality data from HMIS. Additionally, factors such as human resources for health which affect child survival were not included due to lack of spatio-temporal data. The introduction and expansion of District Health Information System version 2 (DHIS2)^129 130^ as part of the HMIS in Kenya and the development of approaches to deal with limitations of routine data^131–134^ will allow incorporation of more variables in future analyses.

Recall and self-report biases were associated with some indicators, especially for longer recall periods, however, this effect was minimised by limiting the recall period to 3 years preceding the survey.^62^ There was selection bias for some indicators because the survey included only the experiences of mothers with a live birth 3 years preceding a survey leaving out mothers with other birth outcomes or those who might have died during pregnancy or delivery. The coverage estimates are not indicative of the quality of interventions received nor do they measure effective coverage which combines the need for, use and quality of the interventions.^12 135^

There was bias due to the modifiable areal unit problem, where results obtained might have been different if data had been aggregated into differently sized spatial units. A small proportion of clusters near county boundaries may have been misclassified because the displacement of cluster coordinates (due to confidentiality) was not accounted for. However, the use of CAR models to smooth estimates across adjacent counties potentially reduced this effect. Some small-scale heterogeneities were masked especially in high sized counties of Northern Kenya and disaggregation of the results to units lower than the counties (subcounties) would improve relevance at county level, however, the precision would reduce drastically. This provides an impetus for a strengthening DHIS2 and in the meantime powering household surveys to be representative at county level and conducting them more regularly.

While exploring inequities across other domains such as disparities across wealth quintiles, urban/rural stratifications or education attainment is important for policy making, however, the focus our work was to explore and describe inequities across geographic areas (counties) for 43 factors. Majority of the other domains including wealth quintiles, urban/rural differences have previously been explored through the standard DHS reports and by Keats and colleagues^21^ in Kenya.

## Conclusion

By harnessing and combining multiple data sources including household sample surveys and population censuses within a geospatial framework, levels and trends of 43 factors associated with child survival were generated between 1993 and 2014 in Kenya. The variation between factors over time was wide and estimates were highly heterogeneous between counties and over time. The marginalised counties that have been left behind should be given priority to address health inequities. The subnational estimates are useful to county planners in the current decentralised system of governance for evidence-based priority setting, a key baseline for monitoring and tracking of interventions within the defined local and global targets such as the SDGs.

## Data Availability

Data are available in a public, open access repository. The full database of household sample surveys, population censuses, malaria surveys and environmental data that support the findings of this study are available open access from online data repositories available to registered users. Integrated Public Use Microdata Series (IPUMS)—https://international.ipums.org/international/index.shtml; Multiple Indicator Cluster Surveys (MICS)—http://mics.unicef.org/; Demographic and Health Surveys (DHS)—https://dhsprogram.com/; Kenya National Bureau of Statistics (KNBS)—http://statistics.knbs.or.ke/nada/index.php/home; Population Health Harvard Dataverse—https://dataverse.harvard.edu/dataverse/population-health.
