# Supplementary material for: Subnational estimates of factors associated with under-five mortality in Kenya: a spatio-temporal analysis, 1993–2014
Source: BMJ Glob Health. 2021 Apr 15;6(4):e004544. doi: 10.1136/bmjgh-2020-004544 (PMC8054106; doi:10.1136/bmjgh-2020-004544)

Additional file 1: Kenya's health context

Figure 1: The Kenya Health context between 1965 and 2018 showing major initiatives that were put in place in Kenya to improve child survival.

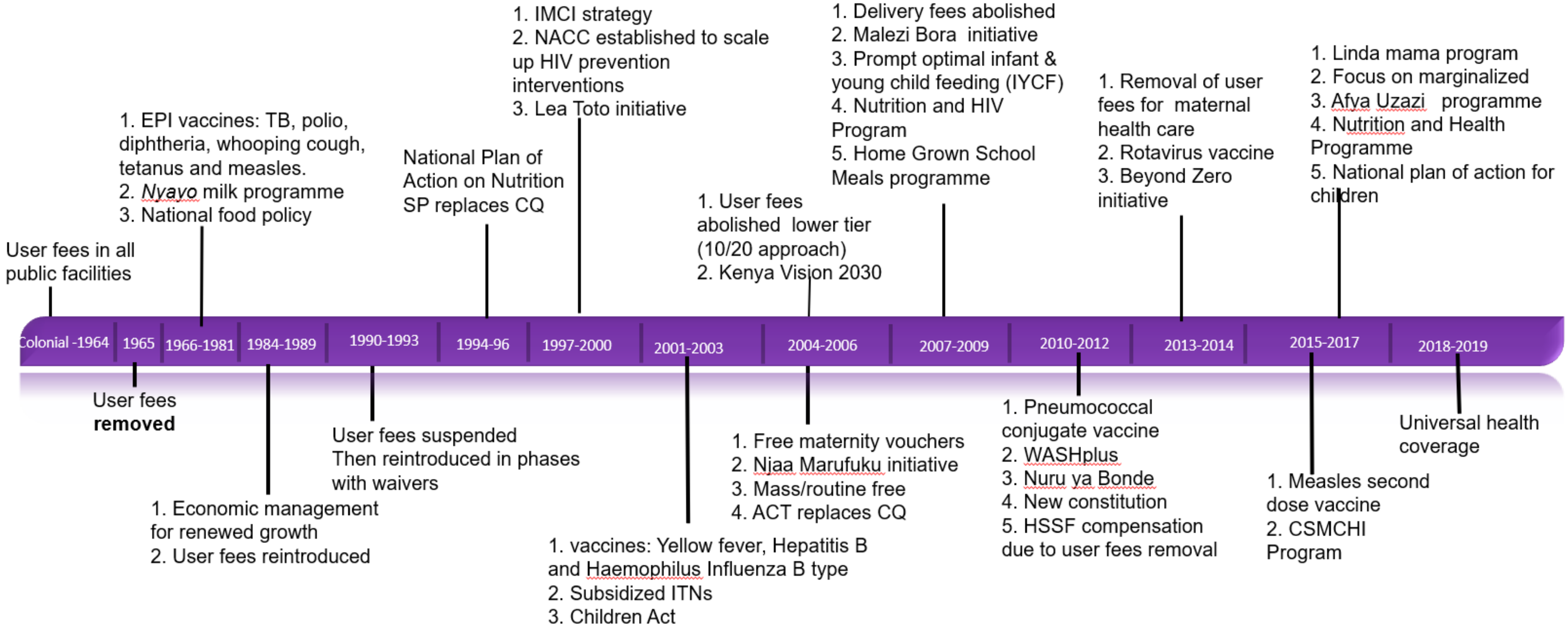

Figure 2: National profile of GDP and major disasters in Kenya including political instability events and conflict (red), flooding (blue) and droughts/failed rains (orange) between 1965 and 2017

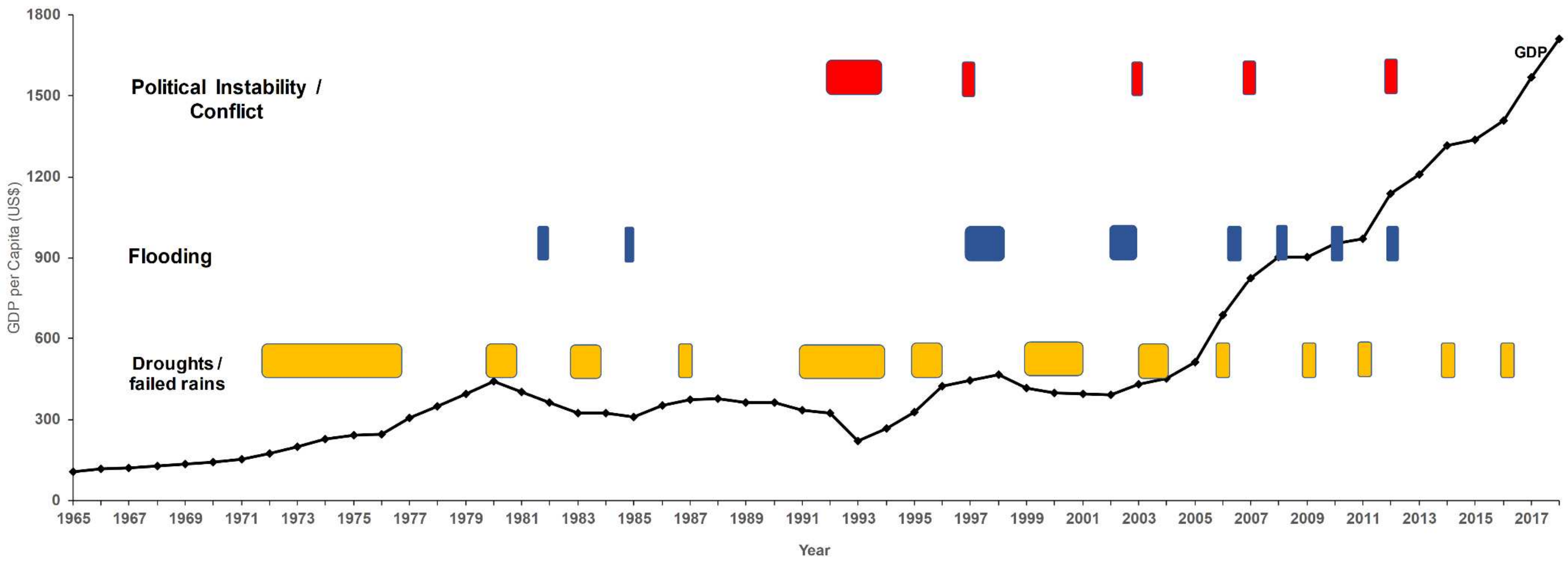

Supplement: Supplementary data [file bmjgh-2020-004544supp001.pdf]
