# Supplementary material for: Subnational estimates of factors associated with under-five mortality in Kenya: a spatio-temporal analysis, 1993–2014
Source: BMJ Glob Health. 2021 Apr 15;6(4):e004544. doi: 10.1136/bmjgh-2020-004544 (PMC8054106; doi:10.1136/bmjgh-2020-004544)
Supplement: Supplementary data [file bmjgh-2020-004544supp002.pdf]

Additional File 2:

Factors associated with child survival including their definition and source of data including household sample surveys or population census.

Group	ID	Variable	Definition	Datasets in reference to Table 1	Time
Environmental factors	1	Rural residency	The proportion of households in rural areas	All census, DHS, WMS, MICS, AIS, KIHBS and MIS	Year of Survey
	2	Precipitation	Annual average precipitation by county between 1990 and 2015	Tropical Rainfall Measuring Mission Multi-Satellite and weather station data.	-
	3	Enhanced vegetation index (EVI)	A vegetation index at 250 spatial resolution averaged to the county level	MODIS -Moderate Resolution Imaging Spectroradiometer-	-
Maternal Factors	4	Maternal education	The proportion of mothers (15-49 years) who had less than primary education at the time of the survey	All DHS, MICS, MIS and KIHBS; WMS 1997, AIS 2007; Census 1989,1999	Year of Survey
	5	Maternal literacy	The proportion of mothers (15-49 years) who can read all or parts of a sentence provided at the time of the survey	All DHS, MIS and KIHBS; WMS 1997; MICS 2007, 2008, 2011, 2013	Year of Survey
	6	Female-headed Households	The proportion of households headed by a female at the time of survey	All census, DHS, WMS, MICS, AIS and KIHBS	Year of Survey
	7	Short birth spacing	The proportion of children with a preceding or succeeding birth interval <24 months	All DHS	Previous 5 years
	8	Use of modern contraceptives	The proportion of women aged 15 to 49 years using any form of modern contraceptive at the time of the survey	All DHS; MICS 2007, 2008, 2011, 2013; WMS 1994	Year of survey
	9	High parity	The proportion of women aged, <30 years with 3 or more children or aged >29 years with 5 or more children	All Census, DHS and MICS; MIS 2015, AIS 2007	
Child factors	10	Underweight	The proportion of children whose weight for age was <=-2 standard deviations at the time of the survey	DHS 1993, 1998, 2003, 2014; MICS 2007,2011; All WMS; KIHBS 2015. The estimates were computed based on the WHO 2006 reference for a well-nourished population	
	11	Wasted	The proportion of children whose weight for height was <=-2 standard deviations at the time of the survey		
	12	Stunted	The proportion of children whose height for age was <=-2 standard deviations at the time of the survey		
	13	Breastfed within the first hour of birth	The proportion of last born <5 years who were breastfed within the first hour after birth	DHS 1993, 1998, 2003 2008, 2014; MICS 2007, 2013; KIBHS 2015	Previous 5 years for DHS, WMS and KIHBS Previous 2 years for MICS
	14	Exclusive breastfeeding	The proportion of lastborn living with the mother and breastfed up to six months at the time of the survey	DHS 1993, 1998, 2003, 2008, 2014; MICS 2007, 2008, 2011, 2013; All KIHBS	
	15	Continued breastfeeding	The proportion of children 6-18 months old who were still being breastfed at the time of the survey.	DHS 1993, 1998, 2003, 2008, 2014; All WMS, MICS and KIHBS	
	16	Low Birthweight (LBW)	The proportion of children weighing < 2500g at birth at the time of the survey among those whose weight was taken	DHS 1993, 1998, 2003, 2008, 2014 MICS 2000, 2011, 2008, 2013: All WMS	
Household factors	17	Poor household	The proportion of households classified as poor or poorer by wealth index constructed using principal component analysis	DHS 1993, 1998, 2003, 2008, 2014; Census 1999,2009; All MICS, MIS and AIS	Year of survey
	18	Improved Sanitation	The proportion of households who have access to flush toilet facilities	All Census, DHS, WMS, MICS, AIS, KIHBS and MIS	
	19	Access to any form of a toilet	The proportion of households who have access to any form of a toilet facility (improved and intermediate)		
	10	Improved water	The proportion of households who have access to piped water for drinking		
	21	Access to wells borehole and piped water	The proportion of households who have access to either piped (improved water) or boreholes/wells water for drinking (Intermediate)		
Infections	22	HIV infection prevalence	The proportion of adults aged 15-49 who were HIV positive during the time of the survey	[1]	Year of survey
	23	Malaria infection prevalence	The proportion of children aged 2-10 years with *Plasmodium falciparum* parasite in their blood.	[2]	Year of survey
Healthcare Utilization	24	At least one antenatal care visit (ANC1)	The proportion of women (15-49) who attended at least one ANC visit by a skilled provider (doctor nurse or midwife) during their last pregnancy in the last three years from the survey time	DHS 1993, 1998, 2003, 2008, 2014; MICS 2007, 2008, 2011, 2013; All AIS and MIS	Previous 5 years for all surveys expect MICS (2 year)
	25	At least four antenatal care visits (ANC4)	The proportion of women (15-49) who attended four or more ANC visit during their last pregnancy in the last three years from survey time	DHS 1993, 1998, 2003, 2008, 2014; MICS 2008, 2011, 2013 AIS 2012; MIS 2015	

	26	Skilled birth attendance (SBA)	Proportion of mothers who were attended by a doctor, nurse, midwife or a community health worker during delivery	All DHS, WMS and MICS; AIS 2012; KIBHS 2015	
	27	Health facility deliveries (HFD)	Proportion of mothers who delivered at a health facility	All DHS, WMS; KIBHS and MICS; AIS 2012	
	28	Diarrhoea treatment-seeking	The proportion of children <5 years who had diarrhoea within the past two weeks before the survey whose treatment was sought from a medical facility	All DHS; MICS 2007, 2008, 2011,2013	Year of survey
	29	Fever/cough treatment-seeking	The proportion of children <5 years who had fever/cough within the past two weeks before the survey whose treatment was sought from a health provider	All DHS and MICS; MIS 2015	
Child health interventions	30	Bacille Calmette–Guérin (BCG)	Proportion of children alive at the time of the survey aged 12-23 months who received BCG vaccine	All DHS, WMS and MICS; KIHBS 2015.	Previous 2 years for all surveys (12-24 months old)
	31	Three diphtheria–tetanus–pertussis vaccinations (DPT3)	The proportion of children alive at the time of the survey aged 12-23 months who received up to the third dose of DPT vaccine		
	32	Three doses of Polio (Polio3)	The proportion of children alive at the time of the survey aged 12-23 months who received up to the third dose of Polio vaccine		
	33	Measles	Proportion of children alive at the time of the survey aged 12-23 months who received measles vaccine		
	34	Fully immunized	The proportion of children aged 12-23 months, alive at the time of the survey who received Polio 3, DPT3, BCG and measles vaccines		
	35	Oral rehydration salts (ORS use)	Proportion of children <5 years old who had diarrhoea within the past two weeks before the survey who received ORS treatment	All DHS, MICS and KIHBS	Year of survey
	36	Vitamin A- children	The proportion of children 6-59 months old who received Vitamin A supplement within six months before the survey	DHS 2003, 2008, 2014; All MICS	
	37	Insecticide-treated bed nets (ITN) use by children	The proportion of children <5 years who slept under an ITN in the previous night before the survey	DHS 2003, 2008, 2014, KIHBS 2005; All MIS	
	38	Recommended antimalarial use	Proportion of children <5 old who had fever within the past two weeks before the survey who received the recommended antimalarial drugs among those given any drug for fever	DHS 2003, 2008, 2014; All MIS and MICS	
Maternal health interventions	39	Tetanus toxoid injection	Proportion of women who received the recommended doses (2) of tetanus toxoid injection during their last pregnancy up to three years since data collection.	DHS 1993, 1998, 2003, 2008, 2014	Previous 5 years
	40	Intermittent preventive treatment in pregnancy (IPTp 1)	Percentage of women (15-49 years) with live birth, three years preceding a survey who during the last pregnancy received at least one dose of SP/Fansidar	DHS 2003, 2008, 2014; All MIS	
	41	IPTp 2	Percentage of women (15-49 years) with a live birth in three years preceding the survey who, during the last pregnancy, received two doses of SP/Fansidar at least one of which was received during an ANC visit		
	42	Iron supplement	The proportion of mothers who took iron tablets/syrup during their last pregnancy	DHS 2003, 2008, 2014	
	43	Vitamin A-mothers	Proportion of mothers who received vitamin A supplement within 2 months after delivery		

The determinants with a recall period of five years preceding the survey (See the column for the year of the survey) were limited to three years due to errors and bias associated with a longer recall period [3].

References

1 NACC. Kenya HIV prevalence estimates. 2020.https://nacc.or.ke/ (accessed 22 Oct 2020).

2 Macharia PM, Giorgi E, Noor AM, *et al.* Spatio-temporal analysis of Plasmodium falciparum prevalence to understand the past and chart the future of malaria control in Kenya. *Malar J* 2018;**17**:340. doi:10.1186/s12936-018-2489-9

3 Ngandu NK, Manda S, Besada D, *et al.* Does adjusting for recall in trend analysis affect coverage estimates for maternal and child health indicators? An analysis of DHS and MICS survey data. *Glob Health Action* 2016;**9**:32408. doi:10.3402/GHA.V9.32408
