# Supplementary material for: Subnational estimates of factors associated with under-five mortality in Kenya: a spatio-temporal analysis, 1993–2014
Source: BMJ Glob Health. 2021 Apr 15;6(4):e004544. doi: 10.1136/bmjgh-2020-004544 (PMC8054106; doi:10.1136/bmjgh-2020-004544)

Additional File 3:

County level estimates of the coverage or prevalence of factors associated with under-five mortality in Kenya: 1993-2014

Figure 1: Rural residency, annual total precipitation and annual average EVI* at the county level between 1993 and 2014 in Kenya.

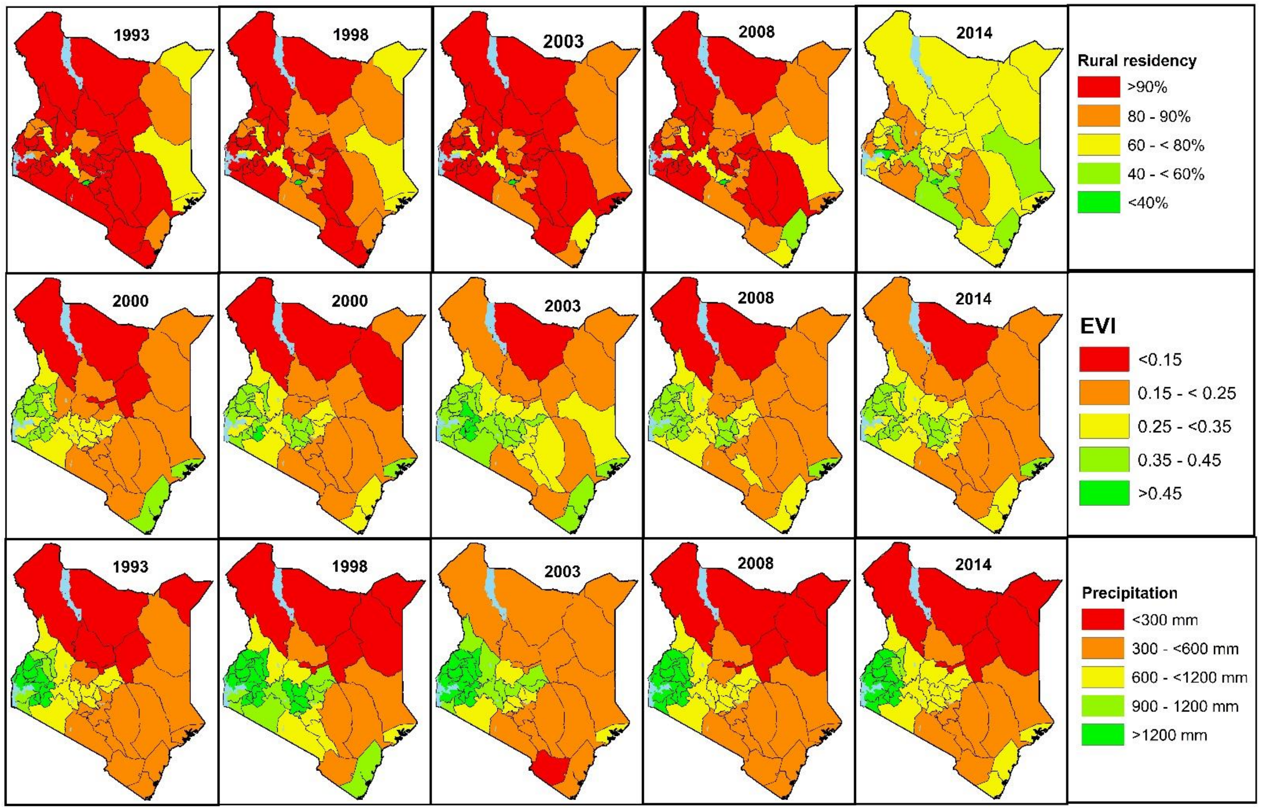

EVI*-enhanced vegetation index

Figure 2: County level trends in maternal education, maternal literacy and the head of a household between 1993 and 2014

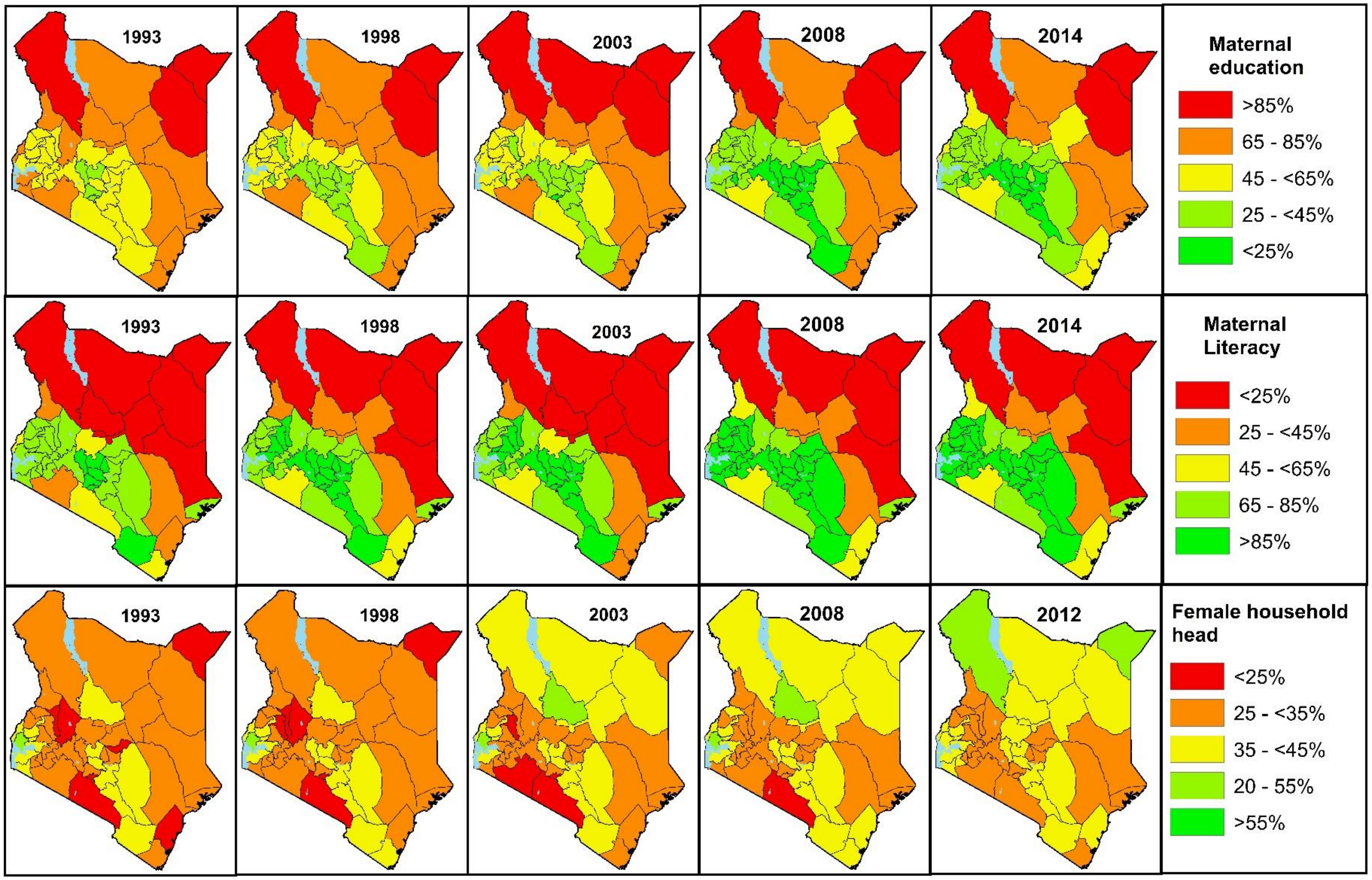

Figure 3: High parity, short birth interval and the use of modern contraceptives at county level between 1993 and 2014 in Kenya.

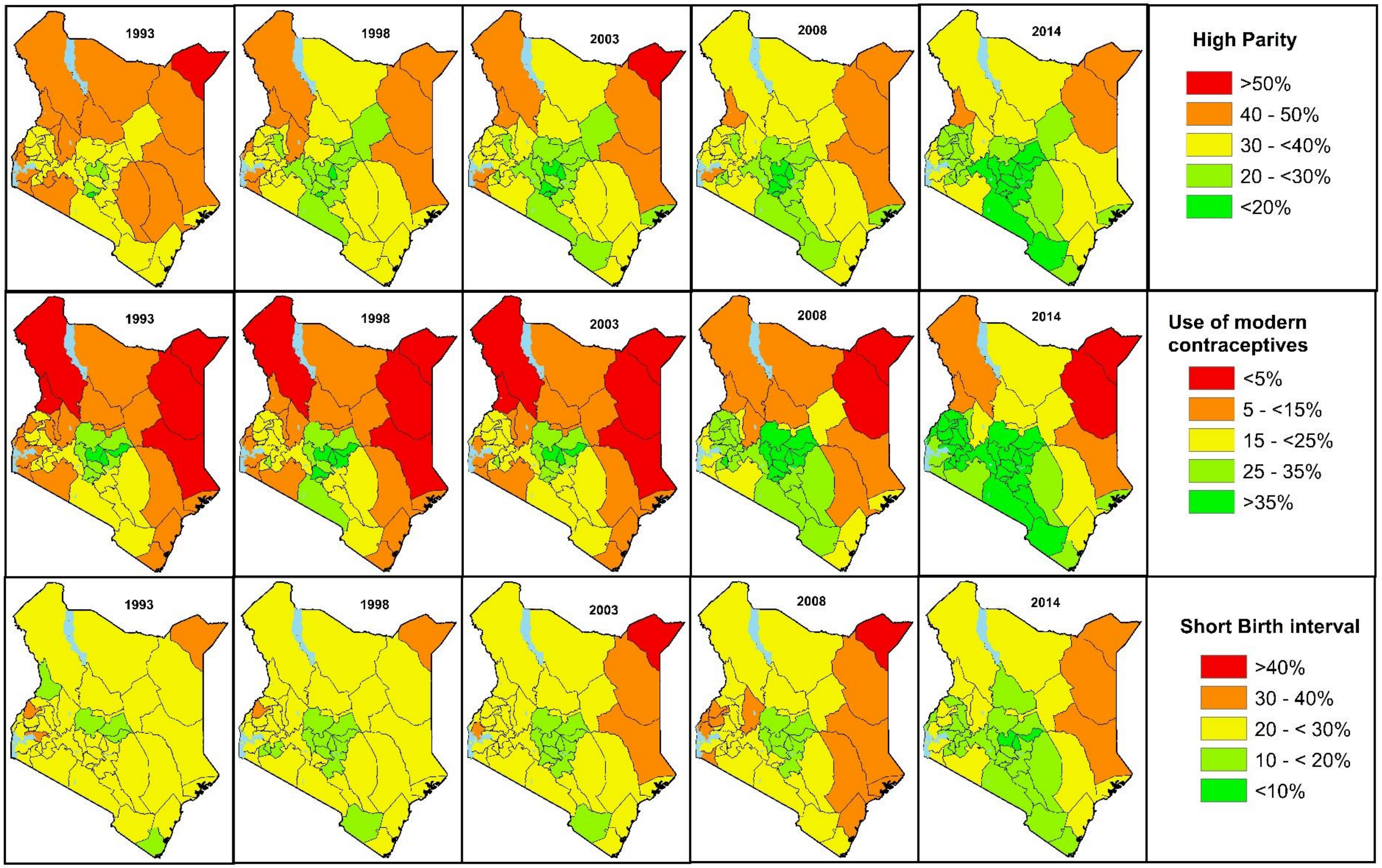

Figure 4: County level prevalence of child growth (stunting, underweight and wasting) between 1993 and 2014 in Kenya.

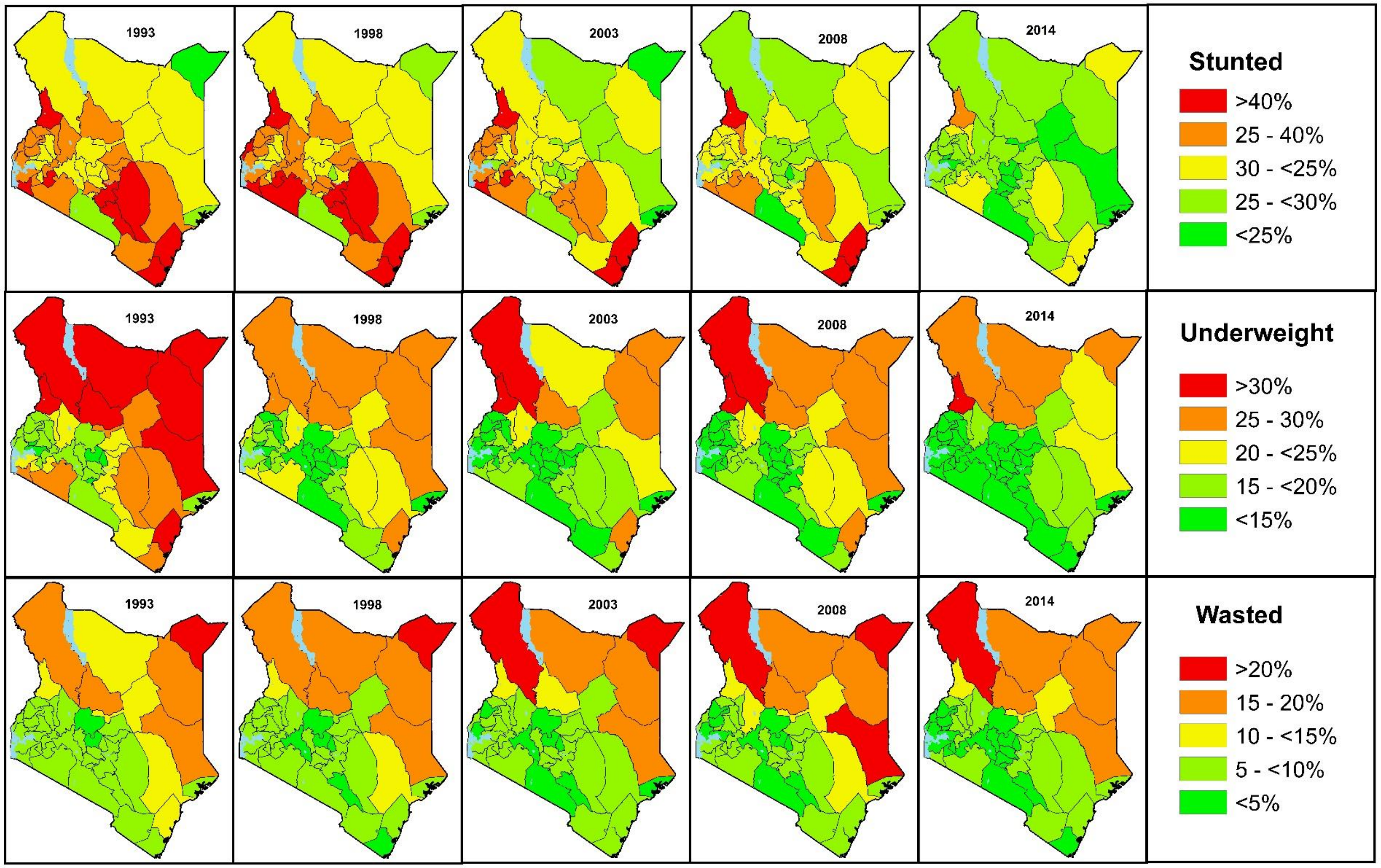

Figure 5: County level coverage of breastfeeding practices and the prevalence of low birth weight in Kenya between 1993 and 2014

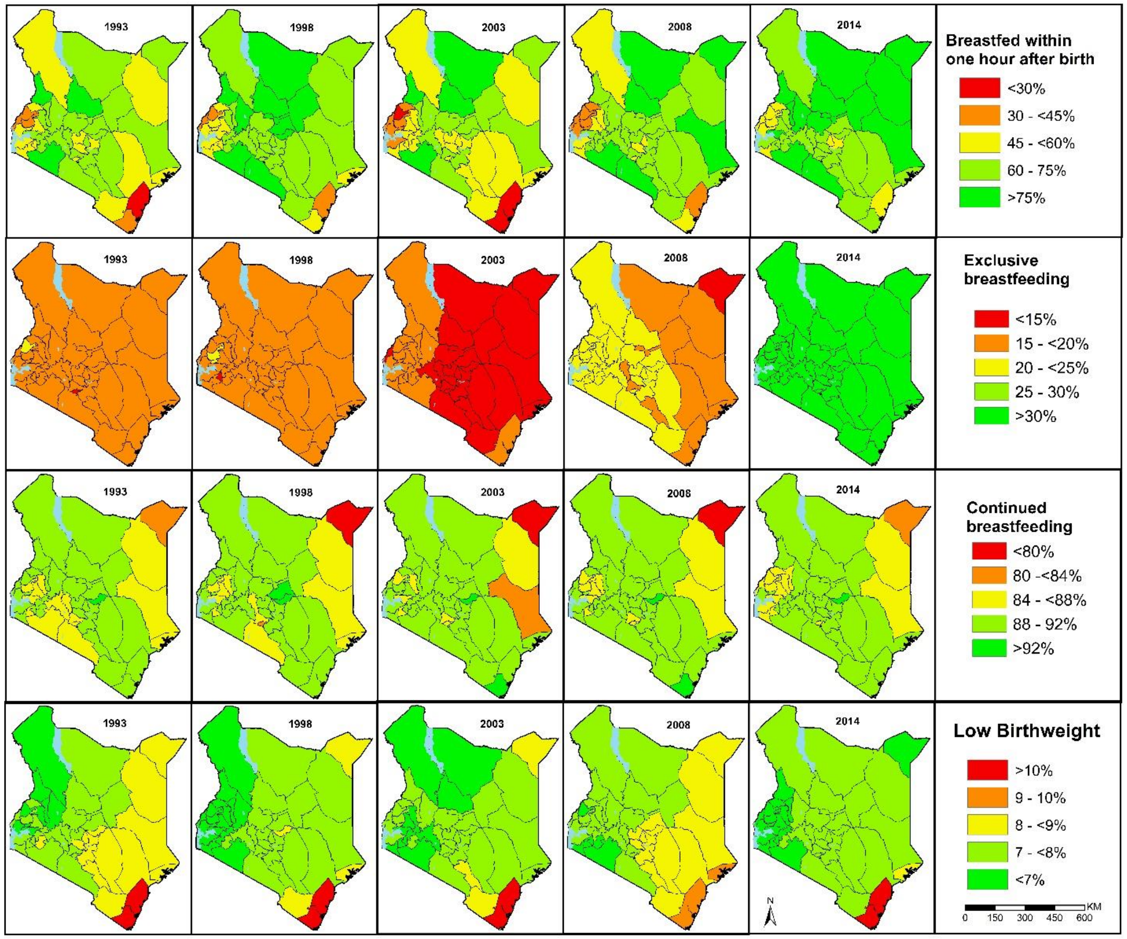

Figure 6: County coverage of access to safe and clean water, sanitation and household wealth in Kenya between 1993 and 2014

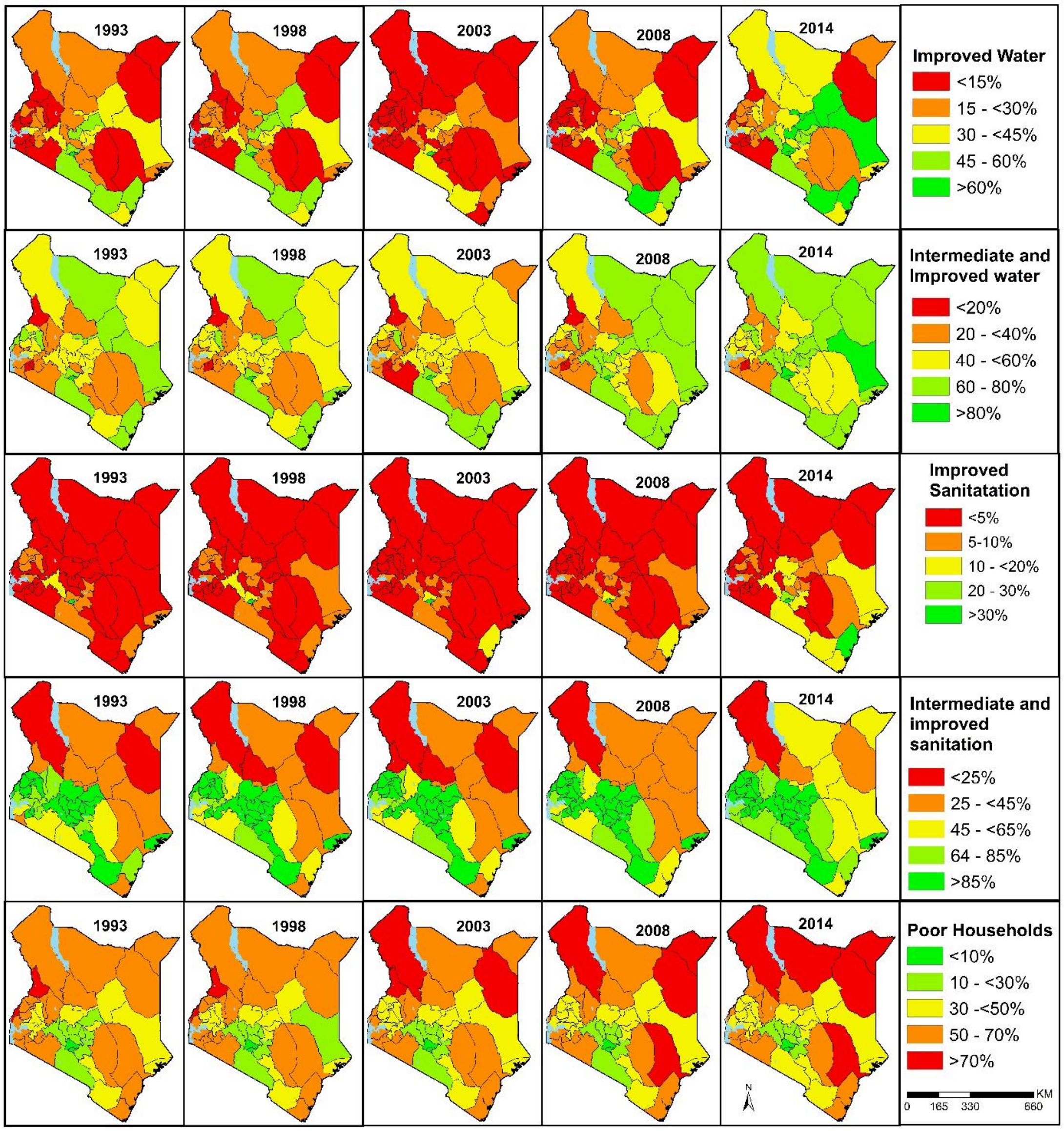

Figure 7: County level prevalence of malaria [1] and provincial level HIV [2] between 1993 and 2014 in Kenya

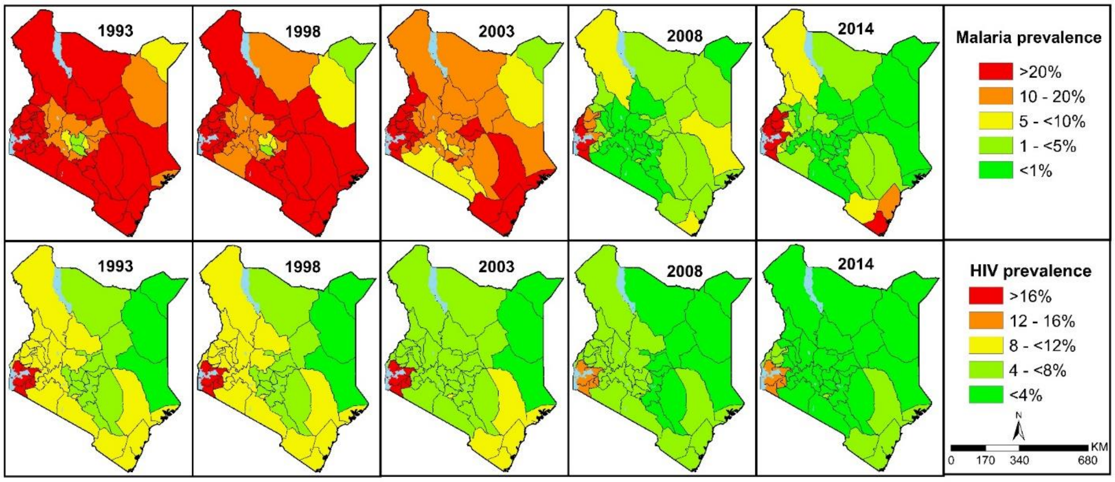

1 Macharia PM, Giorgi E, Noor AM, *et al.* Spatio-temporal analysis of Plasmodium falciparum prevalence to understand the past and chart the future of malaria control in Kenya. *Malar J* 2018;**17**:340. doi:10.1186/s12936-018-2489-9

2 NACC. Kenya HIV prevalence estimates. 2020.https://nacc.or.ke/ (accessed 22 Oct 2020).

Figure 8: County level coverage of at least one ANC visit (ANC1), four ANC visits (ANC4), skilled birth attendance (SBA), health facility delivery (HFD), treatment-seeking for fever and diarrhoea from 1993 to 2014

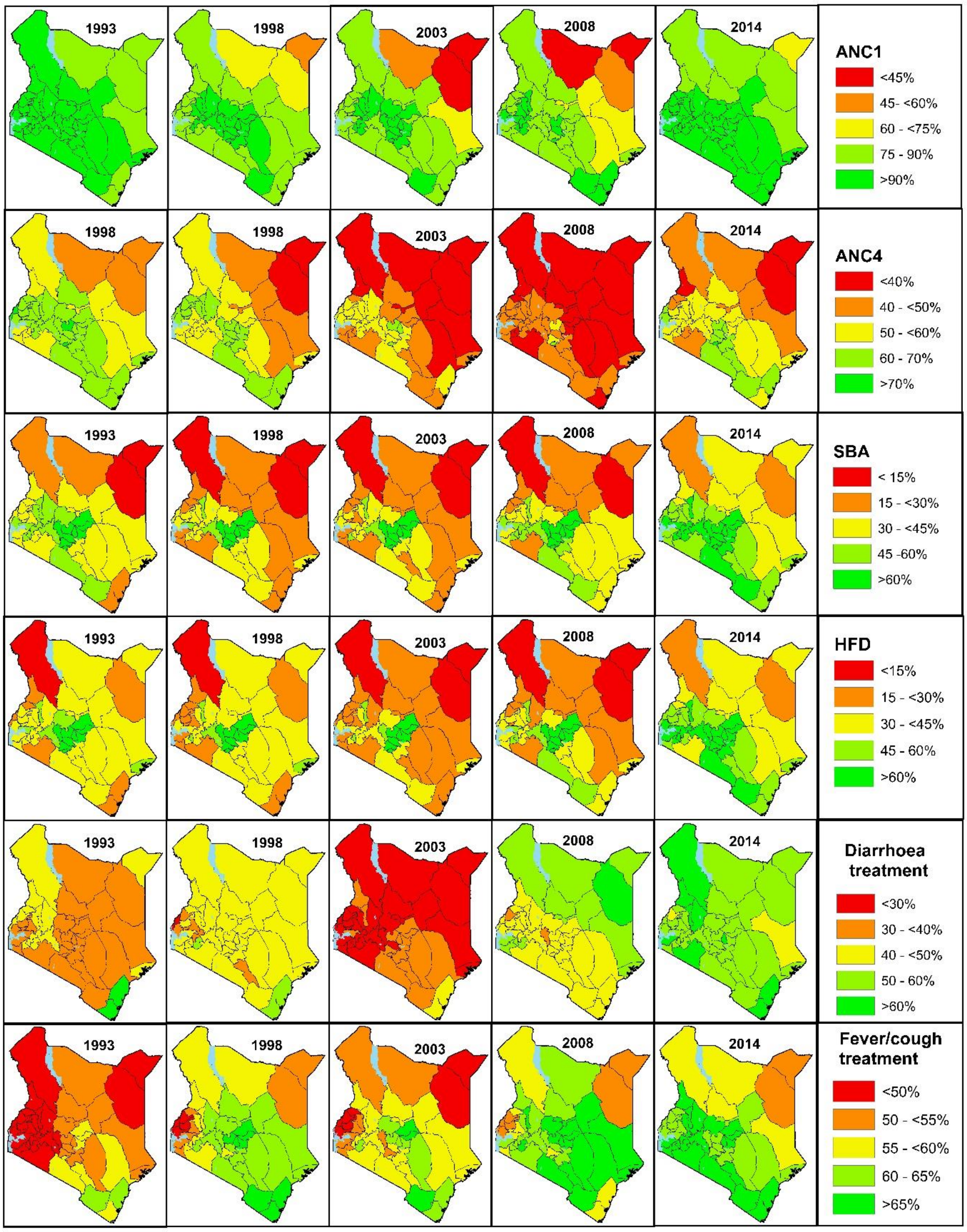

Figure 9: County level immunization coverage for BCG, three doses of DPT, three doses Polio, measles and fully immunized between 1993 and 2014 in Kenya.

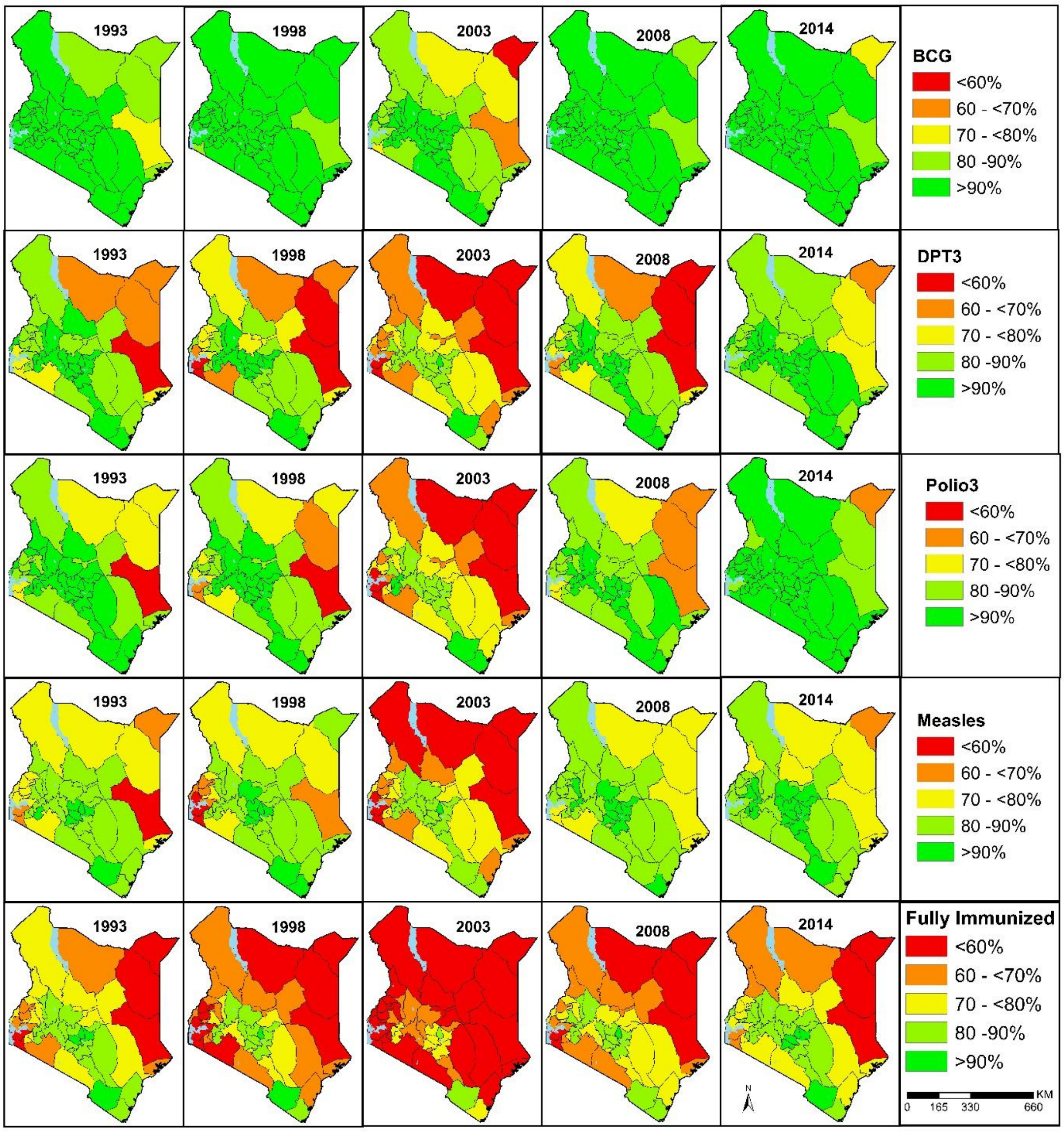

Figure 10: The county level coverage of child health interventions (vitamin A, ITN use, antimalarials and ORS) between 2003 and 2014 in Kenya.

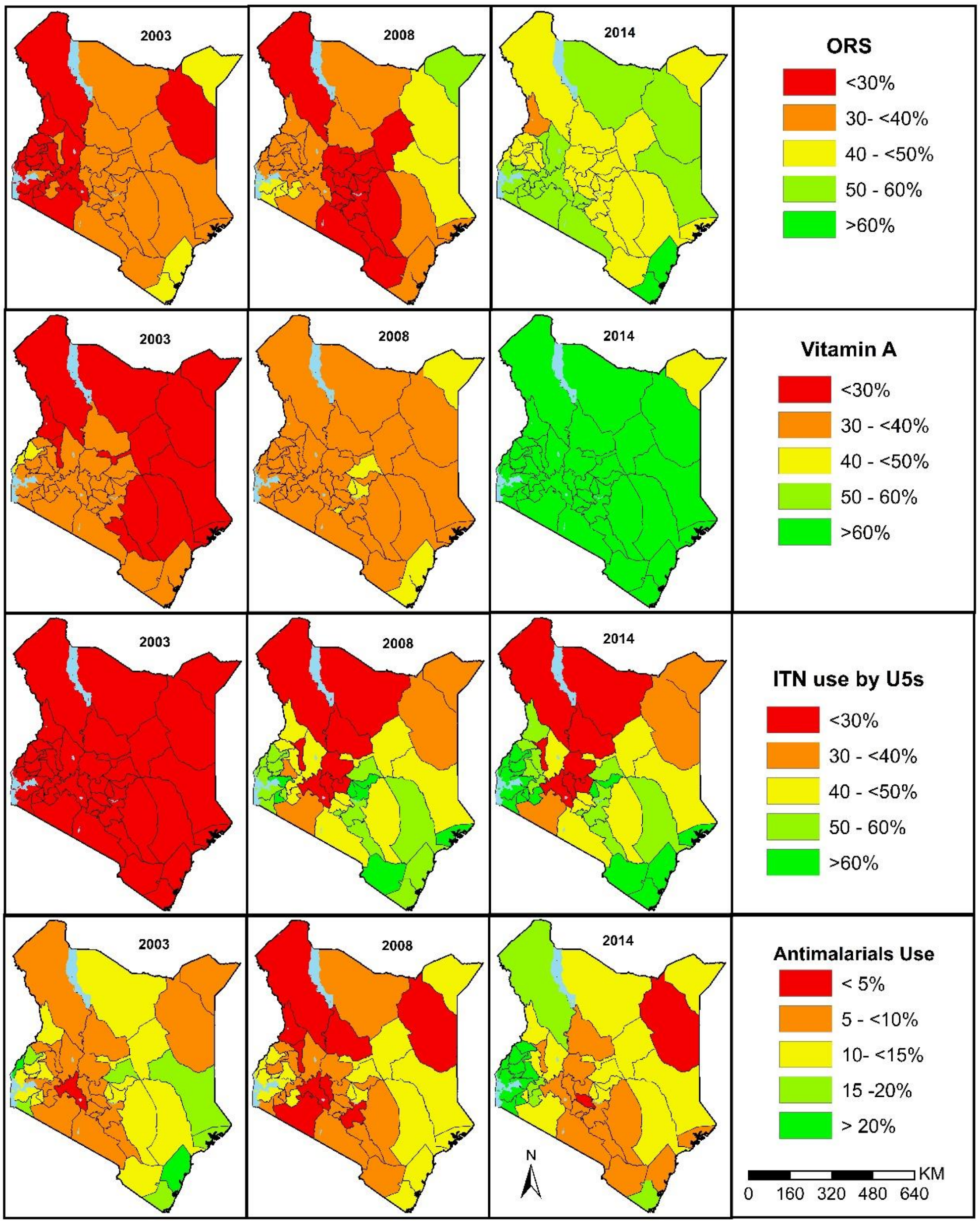

Figure 11: County level coverage of supplements (iron, vitamin A) and Intermittent preventive treatment in pregnancy (one and two doses) between 2003 and 2014 in Kenya.

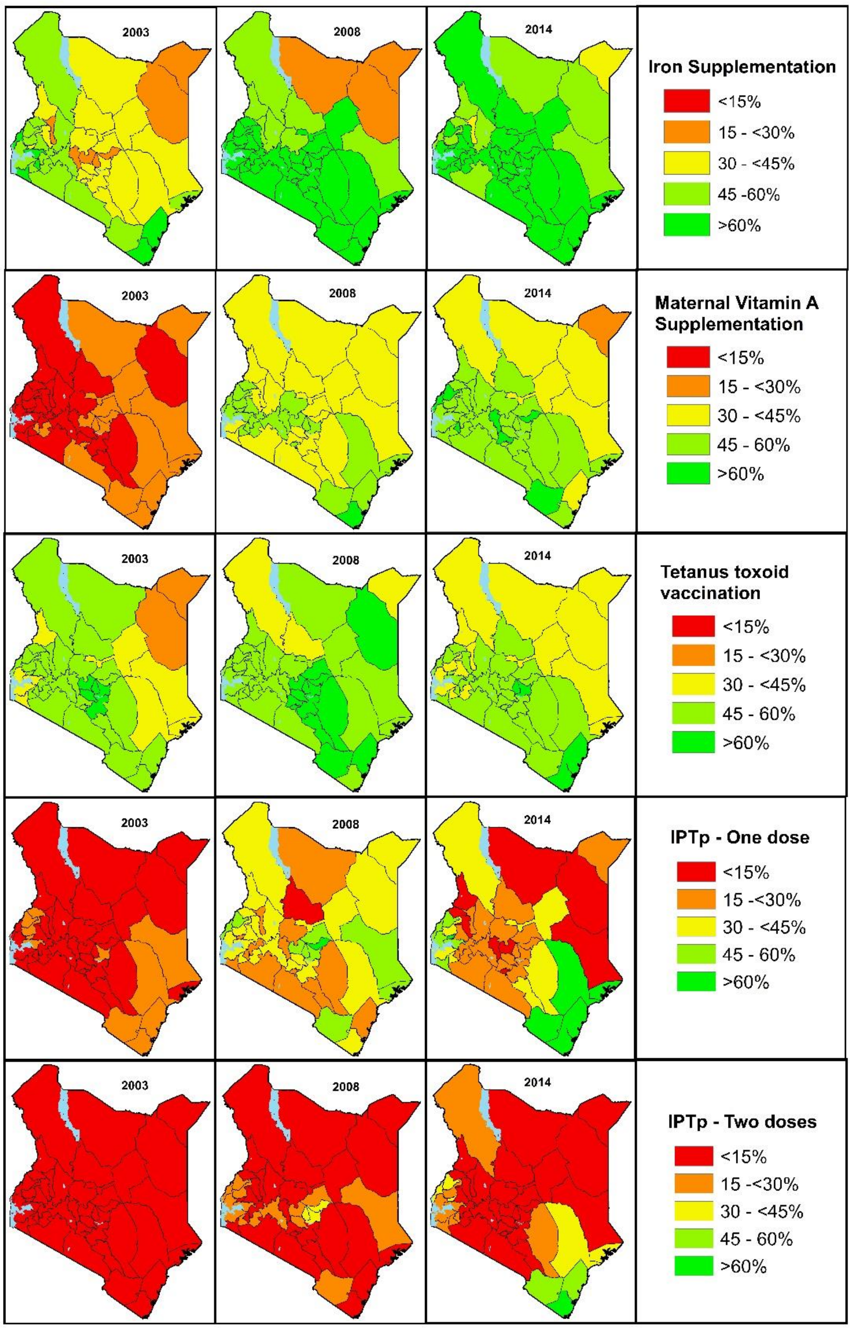

Supplement: Supplementary data [file bmjgh-2020-004544supp003.pdf]
