# Supplementary material for: Subnational estimates of factors associated with under-five mortality in Kenya: a spatio-temporal analysis, 1993–2014
Source: BMJ Glob Health. 2021 Apr 15;6(4):e004544. doi: 10.1136/bmjgh-2020-004544 (PMC8054106; doi:10.1136/bmjgh-2020-004544)
Supplement: Supplementary data [file bmjgh-2020-004544supp004.pdf]

Additional file 4: Validation statistics

The correlation, mean absolute error (MAE) and root mean square (RMSE) from the cross-validation exercise in the spatio-temporal modelling framework. The blue-coloured cells show high correlation (≥0.7), grey -moderate correlation (≥0.5 but < 0.7) and white low correlation (≥0.4 but <0.5).

	ID	Factor	Correlation	MAE	RMSE
Maternal Factors	1	Less than primary school (maternal)	0.95	6.32	9.05
	2	Maternal literacy	0.69	12.2	16.6
	3	Female household head	0.56	6.04	9.05
	4	Short Birth interval	0.65	5.32	7.06
	5	Modern contraceptives use	0.66	5.94	12.12
	6	High parity	0.74	5.8	8.9
Child factors	7	Underweight	0.72	5.51	6.90
	8	Wasted	0.67	2.88	4.05
	9	Stunted	0.55	6.4	8.9
	10	Breastfed within 1st hour of birth	0.70	10.41	12.63
	11	Exclusive breastfeeding	0.99	3.78	5.17
	12	Continued breastfeeding	0.87	4.02	4.91
	13	Low Birthweight	0.42	3.91	4.82
Household factors	14	Poor household	0.61	14.21	20.18
	15	Improved Sanitation	0.89	3.41	6.41
	16	Improved and intermediate sanitation	0.87	7.6	12.6
	17	Improved water	0.85	8.88	13.27
	18	Improved and intermediate water	0.76	12.0	16.3
Healthcare Utilization	19	ANC1	0.64	8.46	13.76
	20	ANC4	0.64	8.66	11.79
	21	Skilled birth attendance	0.76	9.76	12.56
	22	Health facility births	0.90	7.10	10.02
	23	Diarrhoea treatment-seeking	0.61	9.48	12.39
	24	Fever treatment-seeking	0.52	12.13	15.29
Child health interventions	25	BCG	0.65	6.60	12.11
	26	DPT3	0.75	6.36	8.64
	27	Polio3	0.82	8.88	11.13
	28	Measles	0.66	8.76	13.12
	29	Fully immunized	0.64	10.29	13.61
	30	ORS use	0.44	12.38	15.81
	31	Vitamin A- children	0.89	9.80	11.54
	32	Child ITN use	0.82	10.81	14.42
	33	recommended antimalarials	0.55	10.60	14.02
Maternal health interventions	34	Two doses of tetanus toxoid	0.67	10.61	13.02
	35	IPTp1	0.62	13.29	15.88
	36	IPTp2	0.51	10.80	16.56
	37	Iron supplement mothers	0.79	10.41	11.87
	38	Vitamin A mothers	0.83	9.44	11.59
