# Supplementary material for: Subnational estimates of factors associated with under-five mortality in Kenya: a spatio-temporal analysis, 1993–2014
Source: BMJ Glob Health. 2021 Apr 15;6(4):e004544. doi: 10.1136/bmjgh-2020-004544 (PMC8054106; doi:10.1136/bmjgh-2020-004544)
Supplement: Supplementary data [file bmjgh-2020-004544supp005.pdf]

Additional file 5: Corelation coefficients

The correlation coefficients between under five mortality and each of the 43 factors associated with child survival in Kenya. Corresponding p values are presented, brown (not statistically significant) green (statistically significant)

Theme	ID	Factor	Correlation	P value
Environmental factors	1	Vegetation index	0.15	< 0.0001
	2	Precipitation	0.25	< 0.0001
	3	Rural residence	0.03	0.4032
Maternal Factors	4	Less than primary school (maternal)	0.30	< 0.0001
	5	Maternal literacy	-0.18	< 0.0001
	6	Female household head	0.09	0.0047
	7	Short Birth interval	0.42	< 0.0001
	8	Modern contraceptives use	-0.31	0.0299
	9	High parity	0.40	< 0.0001
Child factors	10	Underweight	0.11	0.0005
	11	Wasted	0.08	0.0093
	12	Stunted	0.25	< 0.0001
	13	Breastfed within 1st hour of birth	-0.60	< 0.0001
	14	Exclusive breastfeeding	0.1	0.0013
	15	Continued breastfeeding	-0.1	0.009
	16	Low Birthweight	-0.02	0.6020
Household factors	17	Poor household	0.32	< 0.0001
	18	Improved Sanitation	-0.07	0.0316
	19	Improved and intermediate sanitation	-0.19	< 0.0001
	20	Improved water	-0.27	< 0.0001
	21	Improved and intermediate water	-0.14	0.0006
Infections	22	HIV	0.40	< 0.0001
	23	Malaria	0.65	< 0.0001
Healthcare Utilization	24	ANC1	-0.20	<0.0001
	25	ANC4	0.13	<0.0001
	26	Skilled birth attendance	-0.28	<0.0001
	27	Health facility births	-0.27	< 0.0001
	28	Diarrhoea treatment-seeking	-0.12	0.0001
	29	Fever treatment-seeking	-0.40	< 0.0001
Child health interventions	30	BCG	-0.30	< 0.0001
	31	DPT3	-0.28	< 0.0001
	32	Polio3	-0.21	< 0.0001
	33	Measles	-0.35	< 0.0001
	34	Fully immunized	-0.25	< 0.0001
	35	ORS use	0.06	0.0657
	36	Vitamin A- children	-0.27	< 0.0001
	37	Child ITN use	-0.18	< 0.0001
	38	recommended antimalarials	0.48	< 0.0001
Maternal health interventions	39	Two doses of tetanus toxoid	-0.23	0.0074
	40	IPTp1	0.16	0.0001
	41	IPTp2	0.21	< 0.0001
	42	Iron supplement mothers	-0.16	< 0.0001
	43	Vitamin A mothers	-0.23	< 0.0001
