# Supplementary material for: Subnational estimates of factors associated with under-five mortality in Kenya: a spatio-temporal analysis, 1993–2014
Source: BMJ Glob Health. 2021 Apr 15;6(4):e004544. doi: 10.1136/bmjgh-2020-004544 (PMC8054106; doi:10.1136/bmjgh-2020-004544)
Supplement: Supplementary data [file bmjgh-2020-004544supp006.pdf]

Additional file 5: Comparison between estimates generated in the current work and from other sources

Corelation

Table 1: Corelation coefficients between estimates generated from the current study and estimates from [1–4]

ID	Determinant	Source	Temporal range	Correlation	P value
1	Underweight			0.8575	<0.0001
2	Wasting		2000-2014	0.7728	<0.0001
3	Stunting	[1–3] IHME		0.7475	<0.0001
4	Three doses of DPT			0.8001	<0.0001
5	Improved water			0.7403	<0.0001
6	Skilled birth attendance	[4] Worldpop	2014	0.6547	<0.0001

IHME- institute of Health Measure Evaluation, MAP-Malaria Atlas Project

Ranking of determinants

The high coverage or low prevalence counties were the best performing in 2014 based on estimates from the current study and the comparator with blue coded counties simultaneously identified from the two sets of data. Likewise, for the counties lagging behind (Low coverage or high prevalence counties), analogous interpretation applies.

Table 2: Ranking of estimates generated from the current study and other sources (Table 1 above)

**Determinant**	**High coverage or low prevalence counties**		**Low coverage or high prevalence counties**		**Overall match**
Match	Current study	Table 1	Current study	Table 1	Matched-pairs
Underweight	Nairobi<br>Nyeri<br>Kirinyaga<br>Murang'a<br>Siaya<br>Kisumu<br>Vihiga<br>Kiambu<br>Homa Bay<br>Nyandarua	Nairobi<br>Nyeri<br>Kirinyaga<br>Murang'a<br>Siaya<br>Kisumu<br>Vihiga<br>Kiambu<br>Homa Bay<br>Kisii	West Pokot<br>Turkana<br>Mandera<br>Marsabit<br>Samburu<br>Wajir<br>Baringo<br>Isiolo<br>Tana River<br>Garissa	West Pokot<br>Turkana<br>Mandera<br>Marsabit<br>Samburu<br>Wajir<br>Baringo<br>Isiolo<br>Tana River<br>E-Marakwet	18/20=90%
Wasting	Siaya<br>Vihiga<br>Kirinyaga<br>Nyeri<br>Kakamega<br>Kisii<br>Kisumu<br>Nakuru<br>Kajiado<br>Bungoma	Siaya<br>Vihiga<br>Kirinyaga<br>Nyeri<br>Kakamega<br>Kisii<br>Nyamira<br>Nairobi<br>Murang'a<br>Kiambu	Turkana<br>Garissa<br>Mandera<br>Samburu<br>Wajir<br>Marsabit<br>West Pokot<br>Isiolo<br>Baringo<br>Tana River	Turkana<br>Garissa<br>Mandera<br>Samburu<br>Wajir<br>Marsabit<br>West Pokot<br>Isiolo<br>Baringo<br>Tana River	16/20=80%
Stunting	Nairobi<br>Nyeri<br>Kirinyaga<br>Garissa<br>Mombasa<br>Murang'a<br>Isiolo<br>Kisumu<br>Vihiga<br>Kajiado	Nairobi<br>Nyeri<br>Kirinyaga<br>Garissa<br>Mombasa<br>Murang'a<br>Isiolo<br>Siaya<br>Laikipia<br>Kiambu	West Pokot<br>Kilifi<br>Mandera<br>Kitui<br>Kwale<br>Bomet<br>Trans Nzoia<br>E-Marakwet<br>Narok<br>Baringo	West Pokot<br>Kilifi<br>Mandera<br>Kitui<br>Kwale<br>Bomet<br>Trans Nzoia<br>E-Marakwet<br>Narok<br>Tana River	16/20=80%
Improved (piped) water	Nairobi<br>Mombasa<br>Taita Taveta<br>Nyeri<br>Meru<br>Kiambu<br>Kilifi<br>Isiolo<br>Garissa<br>Kajiado	Nairobi<br>Mombasa<br>Taita Taveta<br>Nyeri<br>Meru<br>Kiambu<br>Kilifi<br>Nakuru<br>Kirinyaga<br>Kwale	Migori<br>Kisii<br>Nyamira<br>Vihiga<br>Busia<br>Homa Bay<br>Wajir<br>Narok<br>West Pokot<br>Tana River	Migori<br>Kisii<br>Nyamira<br>Vihiga<br>Busia<br>Homa Bay<br>Trans Nzoia<br>Nandi<br>Kakamega<br>Siaya	13/20=65%

Three doses of DPT	Kirinyaga<br>Kiambu<br>Bomet<br>Embu<br>Machakos<br>Nandi<br>Kericho<br>Makueni<br>Taita Taveta<br>E-Marakwet	Kirinyaga<br>Kiambu<br>Bomet<br>Embu<br>Machakos<br>Nandi<br>Uasin Gishu<br>Nyamira<br>Vihiga<br>Nyeri	Mandera<br>Wajir<br>West Pokot<br>Garissa<br>Marsabit<br>Migori<br>Turkana<br>Lamu<br>Homa Bay<br>Bungoma	Mandera<br>Wajir<br>West Pokot<br>Garissa<br>Marsabit<br>Migori<br>Turkana<br>Lamu<br>Kajiado<br>Narok	15/20=75%
Skilled birth attendance	Nairobi<br>Murang'a<br>Mombasa<br>Kirinyaga<br>Kiambu<br>Embu<br>Tharaka Nithi<br>Meru<br>Nyandarua<br>Nyeri	Nairobi<br>Murang'a<br>Mombasa<br>Kirinyaga<br>Kiambu<br>Nyamira<br>Kisii<br>Kakamega<br>Busia<br>Vihiga	Wajir<br>Turkana<br>Marsabit<br>Tana River<br>Isiolo<br>Samburu<br>Garissa<br>Mandera<br>West Pokot<br>Trans Nzoia	Wajir<br>Turkana<br>Marsabit<br>Tana River<br>Isiolo<br>Samburu<br>Garissa<br>Lamu<br>Kitui<br>Taita Taveta	12/20=60%

References

1 Kinyoki DK, Osgood-Zimmerman AE, Pickering B V., *et al.* Mapping child growth failure across low- and middle-income countries. *Nature* 2020;**577**:231–4. doi:10.1038/s41586-019-1878-8

2 Mosser JF, Gagne-Maynard W, Rao PC, *et al.* Mapping diphtheria-pertussis-tetanus vaccine coverage in Africa, 2000–2016: a spatial and temporal modelling study. *Lancet* 2019;**6736**:1–13. doi:10.1016/S0140-6736(19)30226-0

3 Deshpande A, Miller-Petrie MK, Lindstedt PA, *et al.* Mapping geographical inequalities in access to drinking water and sanitation facilities in low-income and middle-income countries, 2000–17. *Lancet Glob Heal* 2020;**8**:e1162–85. doi:10.1016/S2214-109X(20)30278-3

4 Ruktanonchai CW, Ruktanonchai NW, Nove A, *et al.* Equality in Maternal and Newborn Health: Modelling Geographic Disparities in Utilisation of Care in Five East African Countries. *PLoS One* 2016;**11**:e0162006.
